# The Adaptive Force as a Potential Biomechanical Parameter in the Recovery Process of Patients with Long COVID

**DOI:** 10.3390/diagnostics13050882

**Published:** 2023-02-25

**Authors:** Laura V. Schaefer, Frank N. Bittmann

**Affiliations:** 1 Health Education in Sports, Department of Sports and Health Sciences, University of Potsdam, 14476 Potsdam, Germany; 2 Regulative Physiology and Prevention, Department of Sports and Health Sciences, University of Potsdam, 14476 Potsdam, Germany; 3 Practice for Integrative Medicine Bittmann, 14467 Potsdam, Germany

**Keywords:** Adaptive Force, maximal isometric Adaptive Force, holding capacity, muscle function, long COVID, post COVID syndrome, muscle weakness, fatigue, neuromuscular control, biomechanical parameter

## Abstract

Long COVID patients show symptoms, such as fatigue, muscle weakness and pain. Adequate diagnostics are still lacking. Investigating muscle function might be a beneficial approach. The holding capacity (maximal isometric Adaptive Force; AFiso_max_) was previously suggested to be especially sensitive for impairments. This longitudinal, non-clinical study aimed to investigate the AF in long COVID patients and their recovery process. AF parameters of elbow and hip flexors were assessed in 17 patients at three time points (pre: long COVID state, post: immediately after first treatment, end: recovery) by an objectified manual muscle test. The tester applied an increasing force on the limb of the patient, who had to resist isometrically for as long as possible. The intensity of 13 common symptoms were queried. At pre, patients started to lengthen their muscles at ~50% of the maximal AF (AF_max_), which was then reached during eccentric motion, indicating unstable adaptation. At post and end, AFiso_max_ increased significantly to ~99% and 100% of AF_max_, respectively, reflecting stable adaptation. AF_max_ was statistically similar for all three time points. Symptom intensity decreased significantly from pre to end. The findings revealed a substantially impaired maximal holding capacity in long COVID patients, which returned to normal function with substantial health improvement. AFiso_max_ might be a suitable sensitive functional parameter to assess long COVID patients and to support therapy process.

## 1. Introduction

Long term sequelae of SARS-CoV-2 infections increasingly challenge medical, social and economic systems worldwide. Different terms are used to define persisting post-infectious symptoms, such as ‘long COVID’, ‘post-COVID syndrome’, ‘post-acute COVID’ or ‘persistent post-COVID’, mostly depending on the duration of symptoms after acute infection. For simplification, the term ‘long COVID’ will be used in the following for patients suffering from symptoms at least 4 weeks after acute infection. Reports on the amount of patients with at least one persistent symptom after SARS-CoV-2 infection range from 10% to 57%, or even up to 87% in hospitalized patients, depending on the time span after acute infection or hospitalization vs. non-hospitalization [1,2,3,4,5,6,7,8,9,10,11,12,13,14,15,16,17,18,19]. Long COVID occurs in 10% to 35% of non-hospitalized patients [1,18], which is most important, since only 5% to 7% of patients are hospitalized [20]. Current data show a lower rate of long COVID after infection with omicron variants than with delta (4.5% vs. 10.8%) [21]. Infection severity is considered to not be a major factor for the development of long COVID [16,22]. According to ‘COVID-19 data Explorer’ from Johns Hopkins University, more than 570 million SARS-CoV-2 cases were confirmed worldwide (Europe, Asia, North America, South America, Africa, Australia) from 22 January 2020 to 28 July 2022. Assuming that 10% of them develop long term sequelae, more than 57 million people suffer or suffered from long COVID. The socioeconomic relevance becomes clear.

The medical community is mainly describing the characteristics of long COVID, but the pathomechanisms or causality are not sufficiently known [6,23]. Furthermore, there is a lack of diagnostic and therapeutic approaches, which are urgently needed to intercept the large amount of sick leave [1,24].

Post-infectious syndromes have been studied for decades and they are known to emerge after different viral infections [25,26,27,28,29,30]. They partly result in myalgic encephalomyelitis/chronic fatigue syndrome (ME/CFS) [11,25,26,30,31,32,33], which is closely connected to long COVID. Symptoms of long COVID range from fatigue, tiredness, muscle weakness, joint/muscle pain, cognitive impairments (‘brain fog’), depression, anxiety, dyspnoea, chest pain/tightness, cough, loss of taste/smell, headache, cardiac symptoms, insomnia, diarrhoea and more [1,2,11,13,14,19,34,35,36]. As can be seen, different systems are involved, including the respiratory, cardiovascular, musculoskeletal, integumentary, gastrointestinal, endocrine and neurological systems [14]. A dysfunction of the autonomous nervous system (ANS) has been discussed as a cause for the symptoms [25,26,30,31,37,38,39]. However, the diagnosis of post-infectious syndromes is difficult and is usually based on a diagnosis of exclusion [24,40]. Patients frequently report that they are not taken seriously by their doctors [34], which even increases the helplessness and anxiety.

A possible supportive diagnostic approach could be to investigate the neuromuscular system since muscle weakness and musculoskeletal pain occur frequently in long COVID patients. Some researchers examined the maximal voluntary isometric contraction (MVIC, e.g., hand grip force) in patients with post-infectious syndromes [41,42,43,44]. Two studies reported non-significant differences between patients and controls regarding the MVIC of the quadriceps femoris muscle (90° knee flexion) or of elbow flexors (90° elbow flexion, maximal supination forearm), respectively [41,42]. Two further studies revealed a significantly reduced hand grip force in ME/CFS [43,44]. However, in Meeus et al., gender effects were not considered [44]. Females were overrepresented in ME/CFS patients vs. controls (96% vs. 62%) [44], which might explain the lower strength. The findings are inconclusive and highlight that common maximal strength assessments might not be that appropriate to investigate muscle dysfunction in post-infectious states.

The Adaptive Force (AF) was inaugurated as a special neuromuscular function, which was found to be sensitive to stimuli [45,46,47,48,49,50,51,52]. The AF characterizes the capacity of the neuromuscular system to adapt to external varying forces in an isometric holding manner [45,46,47,48,49,50,51,52]. It can be assessed by a technical device using pneumatics [45,46,47] or by an objectified manual muscle test (MMT) using a handheld device which measures dynamics and kinematics during the MMT [49,50,51,52]. For the latter, it was shown that the maximal isometric AF (AFiso_max_; maximal holding capacity) was significantly reduced in reaction to negative stimuli, such as unpleasant emotional imagery or odors in healthy participants [50,51,52]. AFiso_max_ immediately decreased by perceiving the negative stimulus and switched back instantaneously to baseline values by applying the positive stimulus. The peak value (maximal AF; AF_max_) was reached during the subsequent eccentric action and was similarly high as for the baseline and positive stimuli. For baseline or under positive stimuli, the muscles remained stable during the whole force increase up to AF_max_ (AFiso_max_ ≥ 99% of AF_max_). Thereby, AFiso_max_ was similar to AF_max_ of unstable muscles. Hence, the maximal force was not influenced by the stimuli but the isometric holding function. In other words, under disturbing influences, the isometric holding capacity broke down to a significantly low level but the maximal strength was not affected. This was interpreted as a high sensitivity of AFiso_max_ with respect to possibly impairing stimuli [49,50,51,52]. Neurophysiological explanations were given previously.

This longitudinal study aimed to investigate the AF in patients with long COVID in a non-clinical setting. For that, AF parameters were assessed at three time points in the course of long COVID: (1) in the long COVID state (pre), (2) after the first treatment (post) and (3) with substantial health improvement (recovery; end). The individual treatments received were not part of the study. It was not a clinical trial; therefore, it was not aimed to measure treatment efficacy. However, the treatments were queried and described to gain an impression of potentially helpful approaches without any claim of evidence.

Based on the current scientific knowledge of AF and of therapeutical experience, the main hypotheses were (1) the holding capacity would be significantly reduced in patients with long COVID and then it would stabilize, thus increase, during the recovery process. (2) AF_max_ would show no significant differences between the time points. (3) AF at onset of oscillations (AFosc) would be significantly higher in long COVID state compared to post and end.

The study provides early data on AF in long COVID patients. If the hypotheses are positively verified, AF might be used as a supportive biomechanical parameter to examine patients with long COVID. Furthermore, AF could help to find the appropriate treatment approach, which will be explained and discussed.

## 2. Materials and Methods

This longitudinal non-clinical study investigated patients in a long COVID state and in the course of their recovery process. Patients were not approached directly. They consulted the practice for Integrative Medicine (Potsdam, Germany; complementary medicine) out of their own personal initiative. If they were diagnosed with post-COVID syndrome or long COVID, they were invited to participate in the study. Regardless of their response, AF data were measured anyway for diagnostic purposes in daily practice. The treatments were neither subject nor part of the investigation. We only aimed to investigate the AF in those patients and its behavior during the recovery process. Therefore, a control group was not included. The measurements took place at the practice of Integrative Medicine and were conducted by researchers from the University of Potsdam (Potsdam, Germany).

### 2.1. Patients

Until July 2022, 37 patients diagnosed with long COVID attended the above-mentioned practice for consultation and the AF was measured initially using a handheld device. The only inclusion criterion was the medical diagnosis ‘post-COVID-syndrome’ or ‘long COVID’, which was received from medical doctors before the patients visited the practice. Exclusion criteria were pre-existing complaints of arm, shoulder, hip or knee of the measured side. Seventeen patients were included in this study since they reported a substantially improved or regained health state by July 2022. The remaining 20 patients were still in therapy or cancelled further therapy because of various reasons (long distance between home and the practice, difficulties in finding appointments, other ongoing treatments/rehabilitation or unknown reasons).

Of the 17 included patients, 14 were female (age: 44.43 ± 14.78 yrs., body height: 168.75 ± 5.23 cm, body mass: 69.93 ± 13.18 kg) and three were male (49.00 ± 7.94 yrs., 187.5 ± 3.54 cm, 94.75 ± 0.35 kg). Further information is given in the Results section (intensity of acute infection, duration from acute SARS-CoV-2 infection to input measurement, symptoms and others).

The study was conducted according to the Declaration of Helsinki and permission from the local ethics committee of the University of Potsdam (Germany) was given (no. 70/2021, date: 16 February 2022). Each participant gave written informed consent.

### 2.2. Questionnaires

The patients filled out two questionnaires. The first one assessed information with respect to acute SARS-CoV-2 infection: duration, medical diagnosis and examination, symptoms and degree of severity (0 = symptom free, 1 = mild, 2 = moderate, 3 = severe but without hospitalization, 4 = hospitalization without intensive care, 5 = hospitalization with intensive care without invasive ventilation, 6 = intensive care with invasive ventilation); as well as concerning long COVID state: period between acute infection and onset of long COVID, periods of improvement, symptoms, diagnosis, medical examinations, experiences with health care, treatments and their effects.

The second questionnaire queried the intensity of common symptoms during long COVID using a scale from 0 (no) to 10 (very strong). The assessed symptoms were fatigue, breathing difficulties, cough, chest pain, chest tightness, memory/concentration problems, headache, muscle pain, fast/strong heartbeat, loss of smell/taste, depression/anxiety, fever, dizziness and post-exertion malaise. Professional and personal stress levels were also queried. The questionnaire was filled out for the following time points: (1) retrospectively for the pre-COVID baseline (before acute SARS-CoV-2 infection), (2) in long COVID state (time of input measurement; pre), (3) 1 day after first treatment (post) and (4) after recovery/with substantial health improvement (output measurement; end).

### 2.3. Handheld Device to Measure the Adaptive Force

**Figure 1:**
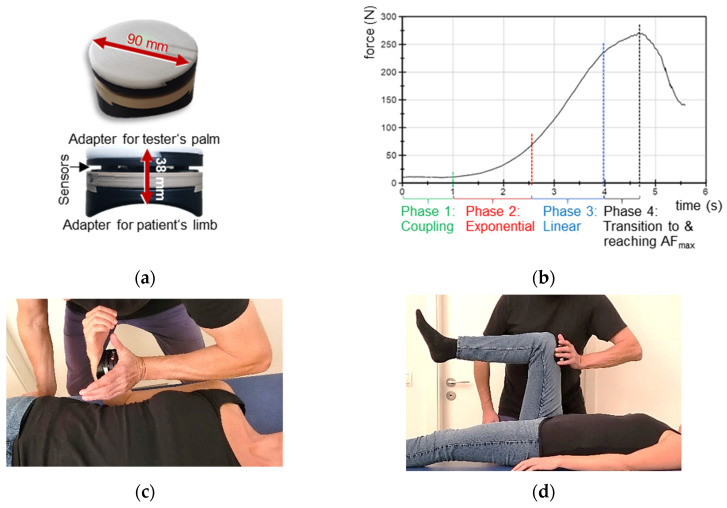
Setting. (**a**) Handheld device; (**b**) force increase during manual muscle test (MMT), including the decisive phases, as was suggested to be optimal by Bittmann et al. [49] and Schaefer et al. [50,51,52]; starting positions of MMTs of (**c**) elbow flexors and (**d**) hip flexors.

The AF of the hip and elbow flexors of one side was assessed by the objectified MMT using the handheld device which was used in previous studies [49,50,51,52]. (Figure 1a; development funded by the Federal Ministry of Economic Affairs and Energy; project no. ZF4526901TS7). It records force and position simultaneously and has proven to be reliable and valid [49]. Strain gauges (co. sourcing map, model: a14071900ux0076, precision: 1.0 ± 0.1%, sensitivity: 0.3 mV/V) and kinematic sensor technology (Bosch BNO055, 9-axis absolute orientation sensor, sensitivity: ±1%) are implemented inside the device. The reaction force between tester and the patient’s limb, as well as the linear accelerations and angular velocity were captured during the MMT. The sampling rate was 180 Hz. The data were buffered, A/D converted and sent via Bluetooth 5.0 to a tablet. An app (Sticky Notes, comp.: StatConsult) saved the transmitted data [49,50,51,52].

### 2.4. Manual Muscle Test to Assess the Adaptive Force: Procedure and Setting

For testing the AF, the MMT in the form of a ‘break test’ was performed [53], since it enables a flexible and time-saving approach. This is especially necessary in fatigued long COVID patients. The MMT aims to assess the patient’s neuromuscular capacity to adapt to an external force increase. It does not test the maximal strength of the patient in the sense of MVIC. MMT characteristics were described previously [49,50,51,52] (Figure 1b). The starting positions of MMT of elbow and hip flexors, including the application of the handheld device, are illustrated in Figure 1c,d (according to [50,51,52]). The patient laid supine. The starting position for the elbow flexor test was 90° elbow flexion and maximal supination of the forearm (Figure 1c). For the hip flexor test, hip and knee angles were ~90° (Figure 1d). The contact with the handheld device was at the distal forearm or thigh, respectively. The contact points were marked and the lever was measured from the lateral epicondyle of the humerus and trochanter major, respectively, to the respective contact point for the standardization of retests. The tester applied a smoothly increasing force (Figure 1b) on the participant’s limb in the direction of muscle lengthening until a considerably high force level was reached. The patient had the task to maintain the starting position in an isometric holding manner for as long as possible. The patient is supposed to react and adapt to the applied force, but the patient was not allowed to push against the tester (for explanation see [50,51,52]). The whole MMT lasted ~4 s.

The MMTs were rated subjectively by the tester: ‘unstable’: the muscle started to lengthen during the force increase, hence, the patient was not able to maintain the isometric position. In that case, the maximal holding capacity (AFiso_max_) was lower than AF_max,_ which was then reached during eccentric muscle action. ‘Stable’: the patient was able to maintain the isometric position until an oscillating force equilibrium occurred at a considerably high force level; in that case, the maximal AF (AF_max_) was reached under isometric conditions (AF_max_ = AFiso_max_). Healthy persons usually show such stable adaptation ($\frac{{\mathrm{AFiso}}_{\max}}{\mathrm{AFmax}}$ ≥ 99%) [50,51,52]. ‘Unclear’: the muscle was neither completely stable nor unstable; slight suspensions were present.

A reproducible force application is a necessary precondition for valid data. Experienced testers are able to perform reliable force profiles over time [49]. Both testers (female, 36 years, 168 cm, 55 kg, 9 yrs. of MMT experience; male, 65 years, 185 cm, 87 kg, 26 yrs. of MMT experience) who assessed the AF of the patients in the present study, had previously proven their ability to test reproducibly [49]. Moreover, the force profiles over time matched precisely between both testers [49].

### 2.5. Procedure

At the first appointment, the patient was examined by means of the MMT by one of the two testers. This tester also conducted all subsequent MMTs of the same patient. Four muscle groups of the lower and upper extremities on both sides were assessed manually (without handheld device), respectively, to obtain an overall impression of the neuromuscular functionality. Then, the input measurements (pre) were performed: AF of hip and elbow flexors of one side was recorded utilizing the handheld device for objectification during the MMT. Patients chose the side to measure, in case of complaints, the complaint-free side was used. Both muscle groups were measured consecutively in alternating order three times, each starting with hip flexors (~1 min resting period between trials). The subjective assessment of the performed MMT by the tester was noted (0 = unstable; 1 = stable, 2 = unclear). Subsequently, the patients received their individual treatment which was not part of the study. Following this treatment (~1 h after input measurements), the AF of hip and elbow flexors was measured again (post) according to the procedure of the pre-measurements. A treatment period of varying duration and number of treatments were prescribed for each patient. During this phase the patients received their individual treatments, which they would have received anyway regardless of the study. The patients were prompted to contact the tester as soon as they felt substantially better or recovered. Then, a final appointment was scheduled for the end measurements (end), that followed the same measuring procedure as for the pre/post measurements. It should be emphasized that no treatment was given at the final appointment prior to the end measurements.

### 2.6. Data Processing and Statistical Analyses

Data processing and evaluation were performed according to Schaefer et al. [50,51,52] in NI DIAdem 2020 (National Instruments, Austin, TX, USA). The recorded data (force and gyrometer signals) were transferred from the measuring app to NI DIAdem. They were interpolated (1 kHz) and filtered (Butterworth, filter degree 5, cut-off frequency 20 Hz). For visualization proposed, the angular velocity was additionally filtered (degree: 3, cut-off: 10 Hz) to smoothen the oscillations. The following AF parameters were captured for further evaluation:

1. Maximal Adaptive Force (AF_max_):

AF_max_ (N) refers to the peak value of a trial. This could have been reached either during isometric or eccentric muscle action.

2. Maximal isometric Adaptive Force (AFiso_max_):

AFiso_max_ stands for the highest force value under isometric conditions. It was defined as the force at the moment in which the gyrometer signal increased above zero (breaking point). This indicated a yielding of the limb and, accordingly, muscle lengthening. If the gyrometer signal oscillated around zero during the entire trial, AF_max_ = AFiso_max_. Thus, the muscle length stayed stable during the whole MMT until the peak force value was reached (stable MMT). If the muscle started to lengthen in the course of MMT, AFiso_max_ was reached during the force increase prior to the peak value. This points out that the position of the limb has to be considered to assess AFiso_max_. In 1 of 256 trials, AFiso_max_ could not be determined because of peculiarities in the curve shape (excluded from evaluation). The ratio of AFiso_max_ to AF_max_ (%) was additionally calculated per trial.

3. Adaptive Force at the moment of onset of oscillations (AFosc):

AFosc (N) characterizes the force at the moment in which oscillations start to appear regularly (onset of oscillations). Previous studies [50,51,52] showed that both interacting partners develop an oscillating force equilibrium, especially during stable MMTs. This was indicated by oscillations which arose in the force signal mostly in phase 3 of MMT (linear increase) showing a clearly distinguishable regular oscillatory behavior. During unstable MMTs, this oscillatory up swing was missing or occurred attenuated on a considerably higher force level. To evaluate AFosc, the force signal was checked for oscillations (force maxima) appearing sequentially during the force increase. If four maxima with a time distance dx < 0.15 s appeared consecutively, the force value of the first maximum was defined as AFosc. Time delta dx < 0.15 s was chosen due to the knowledge that mechanical muscle oscillations occur ~10 Hz [54,55,56,57,58,59,60,61,62,63]. In case no such oscillatory onset occurred, AFosc = AF_max_. In 2 of 256 trials, AFosc could not be clearly determined, hence, they were excluded from evaluation. Ratios of AFosc to AF_max_ (%), as well as AFosc to AFiso_max_ (%) were calculated per trial. The latter is based on previous findings that for stable MMTs, AFosc arose on a lower level than AFiso_max_, and for unstable MMTs, oscillations occurred—if at all—after that breaking point.

4. Slope of force rise:

The slope of force increase prior to the breaking point (AFiso_max_) of all trials was evaluated to control the force application by the tester. This has to be similar between the trials for a valid comparison. The difference quotient m = $\frac{y_{2}-y_{1}}{x_{2}-x_{1}}$ was used to calculate the slope, whereby x refers to time and y to the respective force values. The reference points (time, force) were 70% and 100% of the averaged AFiso_max_ of all of the assessed unstable MMTs of one patient. The decadic logarithm was taken from the slope values (lg(N/s)) since the force rise was exponential. In 11 of 256 trials, the slope could not be determined since oscillations occurred too intensively which would have distorted the slope value.

Arithmetic means (M), standard deviation (SD), coefficient of variation (CV) and 95% confidence intervals (CIs) were calculated per parameter, muscle and time point (pre, post, end) in Microsoft Excel (Microsoft 365, Redmond, WA, USA, Microsoft Corp).

Statistical evaluation was performed with IBM SPSS Statistics 28 (Windows, Armonk, NY, USA, IBM Corp). All parameters (AF_max_, AFiso_max_, AFosc, their ratios and slope) were checked for normal distribution by a Shapiro–Wilk test. In case of normal distribution, a repeated measures ANOVA (RM ANOVA) was used to compare the three time points (pre, post, end). In case the Mauchly test of sphericity was significant, the Greenhouse–Geisser correction was chosen (F_G_). For post-hoc tests, a Bonferroni correction was applied (adjusted *p* values are given by *p*_adj_). Effect size eta squared (η^2^) was given for the RM ANOVA. For pairwise comparisons, the effect size Cohen’s d_z_ was used, which was interpreted as small (0.2), moderate (0.5), large (0.80) or very large (1.3) [64,65]. Since the RM ANOVA is known to be robust against violation of the normal distribution [66,67], a Friedman test was only executed to compare the three time points if more than one dataset (pre, post or end) was not normally distributed (applied for the ratio of AFiso_max_ to AF_max_). The Bonferroni post-hoc test was used for pairwise comparisons (p_adj_) and the effect size Pearson’s r was calculated by r = $\left|\frac{z}{\sqrt{n}}\right|$ in Microsoft Excel. Significance level was α = 0.05.

In addition to the AF parameters, the intensities of the different queried symptoms were evaluated by calculating M and SD. Those values were also compared between the three time points using a Friedman test. Furthermore, the percentage of patients who stated their respective symptoms with an intensity of at least 2 was calculated for descriptive purposes.

## 3. Results

### 3.1. Number of Trials and Subjective MMT Ratings by the Testers

The hip flexors were measured in all 17 patients at the three time points (pre, post, end). The measurements of the elbow flexors were only completed in 14 patients due to reasons such as lack of time, shoulder complaints, too exhausted or similar. In total, 144 MMTs were performed using the handheld device for hip flexors and 118 for elbow flexors. One patient was only tested twice for both muscles because of a lack of time. In two other patients, hip and elbow flexors were only measured twice at pre and post because of tiredness. The hip flexors of another patient were assessed only once at end because of hip pain. In total, 141 valid trials were gathered for the evaluation of hip flexors and 115 for elbow flexors, since technical issues occurred in six trials (3× hip, 3× elbow). In one patient, four trials of hip flexors were performed at the end because the device indicated an error, but the data were nevertheless transferred and, therefore, used for evaluation.

The female tester assessed ten patients and the male tester assessed seven. The number of MMTs rated as ‘unstable’, ‘stable’ and ‘unclear’ by the testers is given in Table 1.

As can be seen, all MMTs (100%) were rated as ‘unstable’ at pre. This reflects that elbow and hip flexors started to lengthen during the force increase, thus, the patients were not able to adapt their muscle length and force adequately in an isometric position during the external force increase. At post and end, the majority of MMTs were rated as ‘stable’ for both muscles (elbow: 92.1% and 97.6%, respectively; hip: 89.4% and 95.9%, respectively). This indicates that the patients were able to adapt to the external force increase in an isometric holding position in the vast majority of MMTs, and the muscles did not yield during the force rise. In total, six MMTs were rated as ‘unclear’ (elbow: 2.6% at post; hip: 6.4% at post, 4.1% at end). This highlights that the MMTs could not be rated as completely stable. The testers mostly described that the muscle showed stronger suspensions than usual for stable MMTs or that the muscle started to yield at a very high force level (especially in comparison to the pre trials). Those subjective ratings should be verified using the data from the handheld device.

### 3.2. Parameters of Adaptive Force in the Course of Long COVID

**Figure 2:**
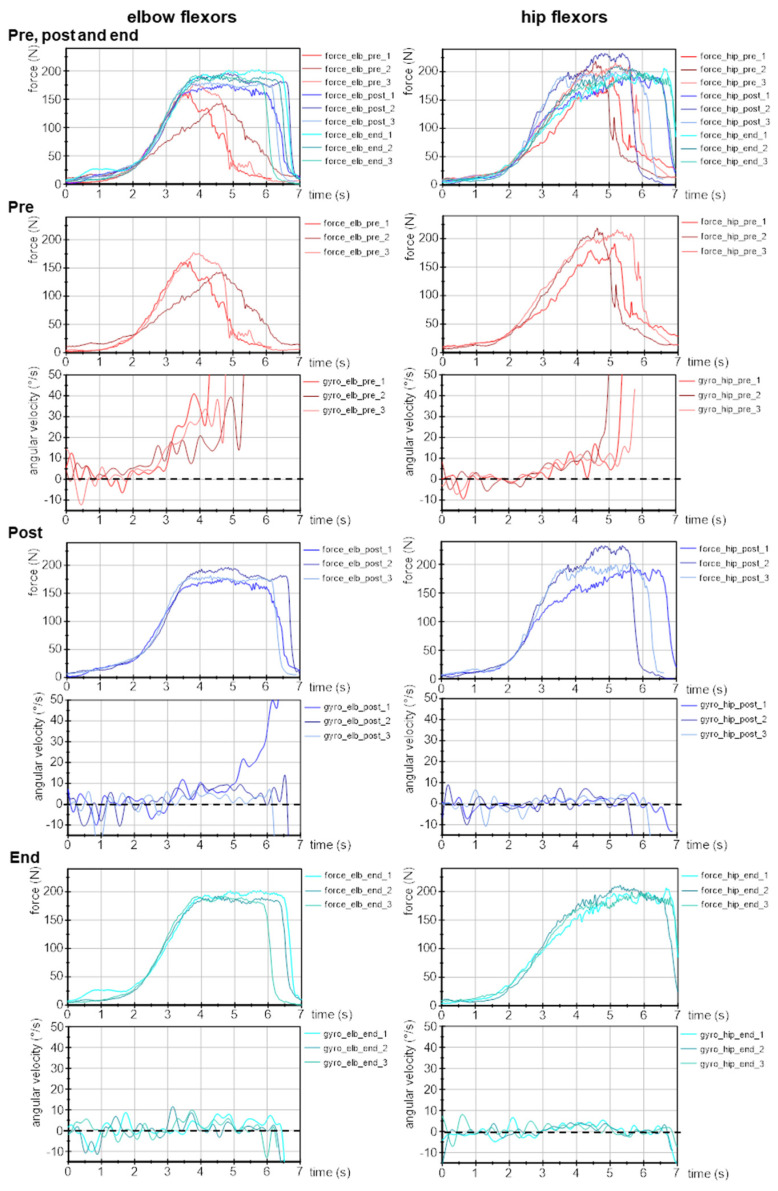
Exemplary signals of force and angular velocity during AF measurements of one female patient (24 yrs., 168 cm, 65 kg) recorded during three MMTs of elbow (**left**) and hip flexors (**right**) at time points pre, post and end (according to Schaefer and Bittmann [68]).

Figure 2 exemplifies the force and gyrometer signals of three MMTs of elbow and hip flexors, respectively, of one female patient (24 years, 168 cm, 65 kg) tested by the male tester at the pre, post and end time points. As can be seen in the uppermost graphs of Figure 2, force rises at pre, post and end can be regarded as similar except for one curve at pre. The single values of each parameter and patient are provided in the Supplementary Materials. Table 2 displays the group averages and statistical results.

#### 3.2.1. Slope of Force Increase

**Figure 3:**
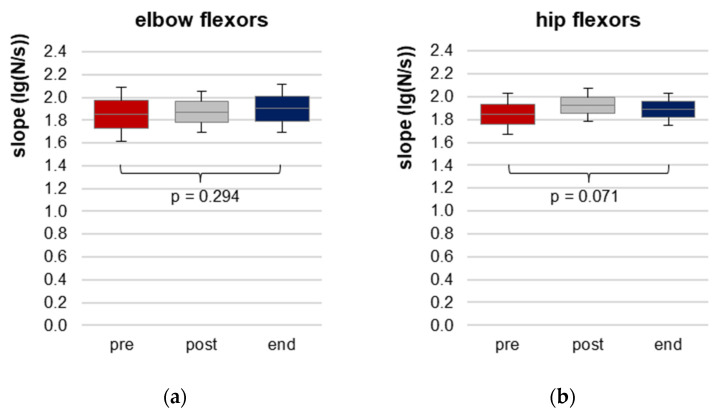
Slope of force increase during the AF assessment. Displayed are arithmetic means, standard deviations (error bars) and 95% CIs of the logarithmic slope (lg(N/s)) of force increase during manual muscle tests for elbow (**a**) and hip flexors (**b**) at each time point pre (red), post (grey) and end (blue). RM ANOVA was non-significant, *p*-values of RM ANOVA are given.

Slope was similar for elbow and hip flexors (Table 2, Figure 3) and did not differ significantly between the three time points, neither for elbow, nor for hip flexors. For the latter, the RM ANOVA was close to significant. The lowest slope was present for pre. Thus, at post and end, the challenge for patients to adapt to the external load can be assumed as even higher. The slope can be interpreted as statistically similar between the time points, which is the prerequisite for comparison of the AF parameters.

#### 3.2.2. Maximal Adaptive Force and Maximal Isometric Adaptive Force

**Figure 4:**
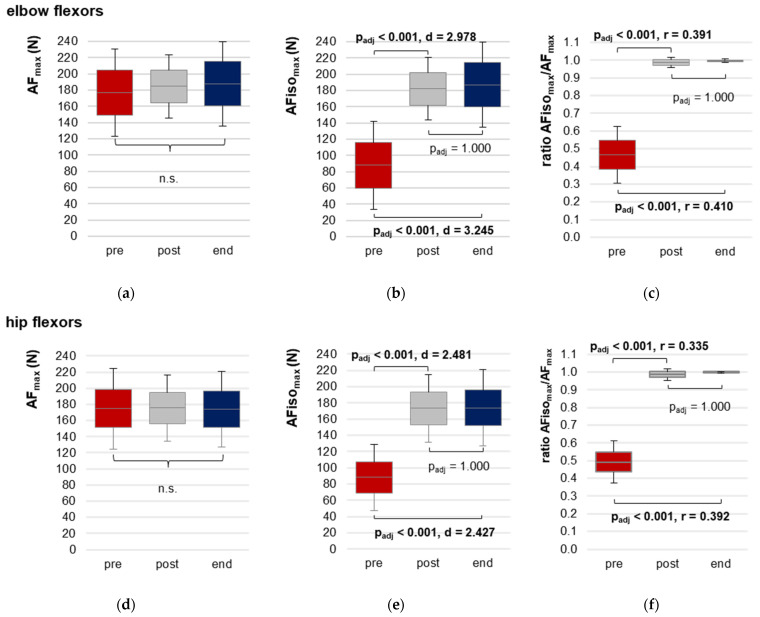
Maximal Adaptive Force and maximal isometric Adaptive Force. Displayed are the arithmetic means, standard deviations (error bars) and 95% CIs of the AF parameters of both muscles at each time point: pre (red), post (grey) and end (blue). Elbow flexors: (**a**) maximal Adaptive Force (AF_max_); (**b**) maximal isometric AF (AFiso_max_) and (**c**) ratio of AFiso_max_ to AF_max_; hip flexors: (**d**) AF_max_; (**e**) AFiso_max_ and (**f**) ratio of AFiso_max_ to AF_max_. The adjusted *p*-values (Bonferroni correction) for the pairwise comparison of the RM ANOVA, as well as of the Friedman test and the respective effect sizes Cohen’s d or Pearson’s r are given in case of significance.

The AF_max_ did not differ significantly between the three time points for both muscles (Table 2, Figure 4a,d). As can be seen in Table 2, AF_max_ was considerably high in the pre measurements. One female patient (outlier) showed an extremely low AF_max_ in the pre trials, with only 61.43 ± 4.86 N for elbow and 67.38 ± 8.66 N for hip flexors. At post, she could increase her AF_max_ immediately to 146.44 ± 22.05 N for elbow and to 162.58 ± 26.01 N for hip flexors. In her case, the AF_max_ at pre amounted to only 42% of AF_max_ at post for elbow and 41% for hip flexors, respectively. This is usually not expected and will be discussed later. The other patients showed AF_max_ values between 145.83 and 295.05 N for elbow and 124.10 and 257.27 N for hip flexors. For timepoints post and end the AF_max_ was considerably high for all patients (Table 2, Figure 4a,d). The AF_max_ at pre related to post amounted averagely 100 ± 14% for elbow and 106 ± 24% for hip flexors, respectively (excl. outlier). Similar for post vs. end with 100 ± 10% for elbow and 102 ± 15% for hip, respectively. Hence, AF_max_ seems to not be appropriate to reflect the testers’ MMT ratings adequately, which showed clear differences between pre and post and pre and end, as well as similar ratings between post and end (Table 1). It has to be mentioned that for all pre trials, AF_max_ was reached during muscle lengthening, whereby for the majority of the post and end trials, AF_max_ arose during isometric muscle action. Therefore, the suggested main parameter to quantify the manually found difference is the maximal force during the isometric muscle action (holding capacity; AFiso_max_).

The AFiso_max_ showed clearly lower values at pre vs. post/end, with a significant main effect in the RM ANOVA (Table 2, Figure 4b,e). The pairwise comparisons revealed significantly lower values for pre vs. post (elbow: t(13) = −11.144, p_adj_ < 0.0001, d = 2.978; hip: t(16) = −10.228, p_adj_ < 0.0001, d = 2.481; one-tailed) and for pre vs. end (elbow: t(13) = −12.140, p_adj_ < 0.0001, d = 3.245; hip: t(16) = −10.007, p_adj_ < 0.0001, d = 2.427, one-tailed). Post vs. end did not differ significantly (elbow: p_adj_ = 1.000; hip: p_adj_ = 1.000).

For elbow flexors, four patients showed an AFiso_max_ below 60 N at pre (range 20.56 to 58.66 N), which has to be considered as extremely low. Hereby, the outlier mentioned above, showed the lowest value. Another patient showed a very high AFiso_max_ = 229.94 N. The others ranged from 62.21 to 156.30 N. All patients showed considerably high AFiso_max_ values at post and end (Table 2, Figure 4).

For hip flexors, three patients showed AFiso_max_ < 60 N at pre (range: 27.36 to 52.00 N), whereby the mentioned outlier again showed the lowest value. The highest AFiso_max_ for pre was 166.73 N, which was reached by the same patient who showed the highest value for elbow flexors. At post and end, AFiso_max_ was considerably high for all patients.

This is mirrored by the ratio of AFiso_max_ to AF_max_ (Table 2, Figure 4c,f). For elbow flexors, it ranged from 23% to 78% at pre, 89% to 100% at post and 97% to 100% at end; for hip flexors, it ranged from 28% to 69% at pre, 87% to 100% at post and 98% to 100% at end. The patients started to lengthen their muscles already at 47 ± 16% of their maximal force (AF_max_) for elbow flexors and at 49 ± 12% for hip flexors in the pre trials. Some patients showed an extremely low ratio in single MMTs. The lowest ratios were 14% for elbow and 15% for hip flexors. In 15 of 36 MMTs (elbow) and 13 of 46 MMTs (hip), the ratio amounted less than 40%. In 11 and 13 MMTs, respectively, it was >60%. The others were in-between 40% and 60%.

At post, already 12 of 14 patients were able to generate at least 98% of AF_max_ for elbow flexors, two patients reached lower values (89% and 96%). It was similar for hip flexors, whereby 14 of 17 patients were able to demand at least 98% of AF_max_, three showed lower values (87%, 93% and 97%). In all end trials, the patients were able to reach 100% of AF_max_ in an isometric holding position, except for two patients who showed values of ~97% or ~98% for elbow flexors and one who reached ~98% for hip flexors.

#### 3.2.3. Onset of Oscillations during Force Increase

**Figure 5:**
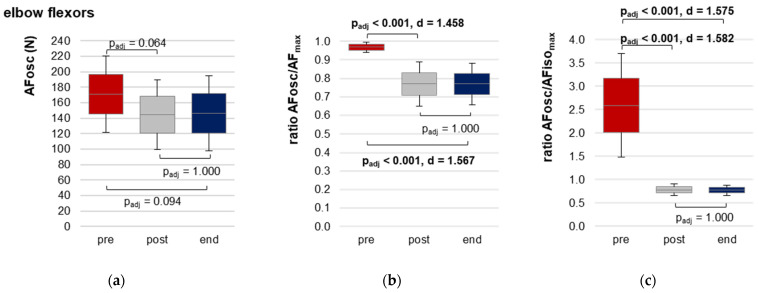

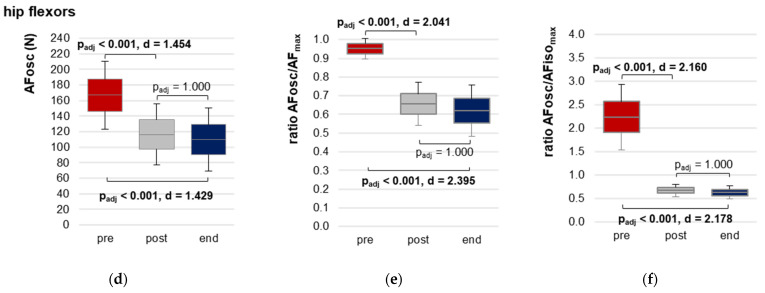
Adaptive Force at onset of oscillations. Displayed are the arithmetic means, standard deviations (error bars) and 95% CIs of AF parameters with regard to the onset of oscillations during MMT for both muscles at each time point: pre (red), post (grey) and end (blue). Elbow flexors: (**a**) AF at onset of oscillations (AFosc); (**b**) ratio of AFosc to AF_max_ and (**c**) ratio of AFosc to AFiso_max_; hip flexors: (**d**) AFosc; (**e**) ratio of AFosc to AF_max_ and (**f**) ratio of AFosc to AFiso_max_. Adjusted *p*-values (Bonferroni correction) and effect sizes Cohen’s d are given in case of significance.

The AFosc revealed a significant main effect in the RM ANOVA for both muscles (Table 2). For elbow flexors, oscillations started at an 18% and 17% higher force level comparing pre vs. post and pre vs. end, respectively. The pairwise comparisons missed significance after the Bonferroni correction (Figure 5a,d). For hip flexors, oscillations occurred at a 43% and 52% higher force level comparing pre vs. post and pre vs. end, respectively. Pairwise comparisons were highly significant (pre vs. post: t(16) = 5.997, p_adj_ < 0.0001, d = 1.454; pre vs. end: t(16) = 5.892, p_adj_ < 0.0001, d = 1.429). Post vs. end did not differ significantly (p_adj_ = 1.000) (Figure 5a,d).

The above-mentioned outlier regarding AF_max_ showed an extremely low AFosc for both muscles at pre with 60.84 (elbow) and 66.48 N (hip). For elbow flexors, AFosc was 100.94 N at post and 100.90 N at end. For hip flexors AFosc was similarly low comparing pre (66.48 N), post (62.88 N) and end (69.89 N). The other patients showed AFosc of elbow flexors in the range of 145.69 to 272.70 N at pre, 62.98 to 217.69 N at post and 52.00 to 236.84 N at end. For hip flexors, it ranged from 116.90 to 242.17 N at pre, from 62.88 to 188.86 N at post and from 29.86 to 191.76 N at end. The between-patients CV for pre, post and end was large with ~31 ± 2% for elbow and ~32 ± 6% for hip flexors. The intraindividual CV was considerably lower with 5.8 ± 2.8% (pre), 12.1 ± 8.3% (post) and 12.8 ± 5.3% (end) for elbow flexors and 6.1 ± 4.1%, 12.3 ± 7.03% and 16.5 ± 15.1%, respectively, for hip flexors.

The ratio of AFosc to AF_max_ was clearly and significantly higher at pre vs. post and pre vs. end for both muscles (Table 2, Figure 5b,e) (elbow: pre vs. post: t(13) = 5.455, p_adj_ < 0.0001, d = 1.458; pre vs. end: t(13) = 5.863, p_adj_ < 0.0001, d = 1.567; hip: pre vs. post: t(16) = 8.306, p_adj_ < 0.0001, d = 2.014; pre vs. end: t(16) = 9.876, p_adj_ < 0.0001, d = 2.395). No significant differences were present comparing post vs. end.

Displayed by the ratio of AFosc to AFiso_max_, the oscillations arose consistently after the breaking point (AFiso_max_) at pre. At post and end, they occurred before AFiso_max_ (Table 2, Figure 5c,f). Only in one MMT of elbow flexors at post, the oscillations arose just with AFiso_max_ (AFosc/AFiso_max_ = 100%). The RM ANOVA revealed a significant main effect for both muscles (Table 2). Pairwise comparisons were highly significant for pre vs. post (elbow: t(13) = 5.918, p_adj_ < 0.0001, d = 1.582; hip: t(16) = 8.905, p_adj_ < 0.0001, d = 2.160) and pre vs. end (elbow: t(13) = 5.892, p_adj_ < 0.0001, d = 1.575; hip: t(17) = 8.979, p_adj_ < 0.0001, d = 2.178). Post vs. end showed no significant differences (p_adj_ = 1.000 for both muscles).

### 3.3. Patients Characteristics Regarding Long COVID

The patients’ professions were teachers/educators (6) students/trainees (2), IT specialist (1), editor (1), lawyer (1), filmmaker (1), social insurance clerk (1), physiotherapist (1), business economist (1), manager of a coronavirus test center (1) and pensioner (1). From the 16 employed patients, 14 were unable to work because of long COVID at the first appointment (pre), one had just started to work again and one had no sick leave at all. At timepoint end, eight of the 14 incapacitated patients were working again and six wanted to return to work again soon. One was still on sick leave.

The acute SARS-CoV-infection lasted on average 15.29 ± 9.40 days (range: 7 to 40, n = 17). The median of acute infection severity was 2.25 (n = 16). One patient was admitted to hospital due to vertigo, another because of a suspected heart attack (nevertheless, she rated the intensity with 1). Overall, infections could be interpreted as mild.

The duration from acute infection to input measurement (pre) was on average 274.88 ± 210.70 days (range: 32 to 688). From pre to end, the duration amounted to 71.06 ± 44.43 days (range: 26 to 166 days). The patients had on average 3.29 ± 1.79 (range: 1 to 7, n = 17) treatment appointments at the practice from pre to end. At end, four patients were completely recovered and required no further appointments. Thirteen patients reported to feel sustainably better but wanted to receive further treatments. For 10 of those 13 patients the therapy phase was completed after an average of 2.80 ± 1.99 further treatments. Three patients were still in therapy (July 2022), since they had not regained their full quality of life back or they wanted to stabilize their health further, especially with regard to mental stability.

The patients reported a large variety of symptoms in the long COVID state, which were not all regarded in the questionnaire. Beyond the queried symptoms, the patients reported recurrent ‘crashes’, (muscle) weakness, joint stiffness, limb heaviness, feeling of whole body paralysis, brain fog to black outs, aphasia, forgetfulness, slowed reaction, sensitivity to stimuli, such as light/noise, hypersomnia or sleeping problems, vertigo, nausea, diarrhea, sore throat, ague, strong sweating, impaired vision, olfactory hallucination, body aches (back/shoulder/neck/heart/lung/tooth/eyes), tingles in the nerves/limbs/head/tongue, cold hands/feet, increased convulsion tendency, internal vibrating, inner restlessness, being phlegmatic, high level of irritability, fast overload, mental imbalance, depression, tachycardia, extrasystoles, hair loss, eczema, herpes, reactivated Epstein–Barr virus infection and tinnitus.

**Figure 6:**
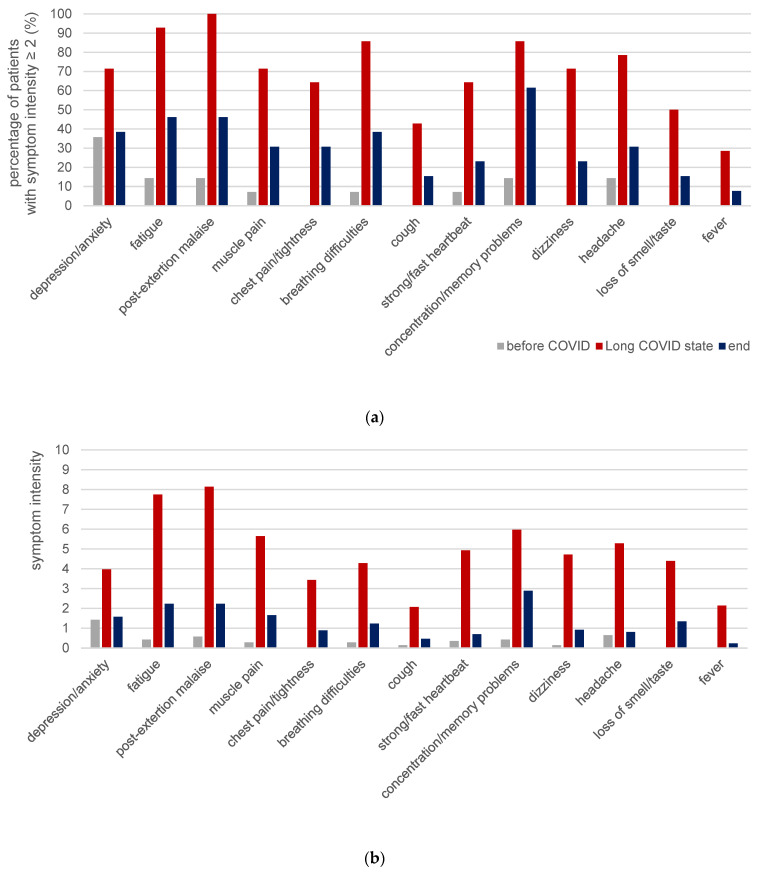
Symptom intensity. (**a**) Percentage of patients who rated the symptom intensity with ≥2. (**b**) Arithmetic means of the symptom intensity on the scale from 0 (no) to 10 (very strong). Both are given for each symptom and time point: before COVID (grey, n = 14), during long COVID state (red, n = 14, corresponds to pre measurements), and end (blue, n = 13, corresponds to end measurements).

Figure 6a illustrates the percentage of patients who stated the respective symptoms with an intensity of at least 2. As can be seen, no patient reported to have chest pain/tightness, cough, dizziness, loss of smell/taste or fever with such an intensity before COVID. However, the majority of symptoms was already present in some patients—at least slightly—before COVID infection (7.14% to 35.71%, n = 14). Depression/anxiety showed the highest percentage before COVID. This is in line with the statements regarding job-related and personal stress before COVID (Table 3), which were rated with an intensity of ≥2 in 90.91% and 100%, respectively (n = 11). Those values declined in long COVID state to 85.71% (n = 14, three patients did not check the boxes because of sick leave) and 88.24% (n = 17), respectively; at timepoint end, they amounted to 30.33% and 50.00% (n = 12), respectively.

Absolute symptom intensities are displayed in Table 3 and Figure 6b. The Friedman test was significant for each symptom. From ‘before COVID’ to ‘long COVID state’, the intensities increased significantly for each symptom (*p* < 0.001 to 0.024), except for fever (*p* = 0.202). In long COVID state, all patients reported to suffer from fatigue, post-exertion malaise and breathing difficulties with an intensity of at least 1. Most prominent were post-exertion-malaise and fatigue with an intensity of 8.1 and 7.8, respectively. The other symptoms did not occur in each patient, whereby fever was the rarest (four patients).

Comparing ‘long COVID state’ vs. end, the intensity of all symptoms declined, most of them significantly (*p* < 0.001 to 0.031), except for fever (*p* = 0.281) and loss of smell/taste (*p* = 0.062) (Table 3, Figure 6).

The symptom intensities did not differ significantly between ‘before COVID’ and end (*p* = 0.202 to 0.922), whereby for concentration/memory problems, significance was just missed with *p* = 0.050. The latter was still present in 11 of 13 patients (three of them stated an intensity of 1, one patient of 9). Another patient stated that an elevated temperature was partly still present at timepoint end when he was physically active (rated fever with intensity 3); after one further treatment this was resolved, too.

Although the individual treatments were not part of the evaluation, they should be reported briefly. Fifteen of 17 patients filled out the questionnaire with respect to their diagnosis and therapies prior to the first appointment. At least seven patients had large diagnostic assessments in centers or rehabilitation facilities for long COVID, the others stated to have received assessments from medical specialists (pulmonologists, internists and similar). The initiated treatments ranged from rehabilitation measures, such as physiotherapy including lymph drainage, manual therapy/massage, reflective breathing therapy, hot role and exercise therapy to (hyperbaric) oxygen therapy, ergotherapy, psychotherapy or psychological guidance, behavioral therapy, speech therapy, concentration training, pharmacological approaches (antibiotics, cortisone, asthma inhaler) to dietary changes. The most common advice from medical specialists was ‘pacing’. According to the patients’ statements, this was very frustrating. Four patients stated that they received no advice or arranged therapy from medical specialists. They were told to rest or were not taken seriously. The majority of patients reported having the impression that conventional medicine had no treatment approach and some reported that medical specialists were ‘clueless’. Others stated that as soon as no somatic reason for their condition could be found, the patients were diagnosed with a psychosomatic disorder. Nevertheless, some patients reported positive effects regarding reflective breathing therapy and for psychological guidance to cope with the condition. Regarding physiotherapeutic measures, the effects varied widely. Some patients reported at least a supporting effect regarding manual therapy which helped to reduce some pain in the short-term. Others stated that lymph drainage worsened the condition. Some also stated that exercise therapy helped for their musculature and cardiovascular system, however, others reported a deterioration after low-intensity exercise therapy.

The mentioned helplessness and—if at all—mostly short-term supportive therapies led the patients to try interventions on their own initiative. Those included supplements (mostly vitamins, trace elements), walks, relaxation techniques or meditation, planning of daily routine, concentration training and rest. At least two patients went to naturopaths or specialists in traditional Chinese medicine. However, none of those measures led to the desirable improvement of their health condition. That is why the patients were seeking other approaches and made an appointment at the practice, where the AF measurements took place. Two of the 17 patients were transferred from a pulmonologist, the others came via other ways. The patients were still partly undergoing therapies elsewhere. The additive treatment at the practice for integrative medicine involved an individual approach based on the muscular holding capacity. Some regularities regarding the applied treatments were found. For each patient, an individualized pulsed electromagnetic field therapy (PEMF) was applied. Based on several studies [31,69,70,71,72], an influence on the ANS is assumed. For a single case, the PEMF therapy in long COVID was recently described [68]. Moreover, 11 of 17 patients were treated for mental stress (persisting or post traumatic situations) using an individualized treatment approach. The lymphatic system was treated in seven of 17 patients using manual methods, as well as individualized PEMF. In some cases, osteopathic and chiropractic techniques for the cranial and/or the musculoskeletal system were applied.

## 4. Discussion

The present pilot study investigated the AF of elbow and hip flexors via an objectified MMT in patients with long COVID at three time points: during long COVID state (pre), after the first treatment (post) and after recovery/substantial health improvement (end). The additionally received individual treatments of the patients were not part of the study and were only included descriptively. The evaluation of the slope of applied force rise by the tester revealed a non-significant difference between the three time points. Therefore, the results are based on reproducible force increases and can be regarded as valid. The results supported the hypotheses and will be discussed with regard to different physiological and practical aspects.

### 4.1. Comparison of the Subjective Ratings of the Manual Muscle Test and Measured AF

The MMT was comprehensibly criticized to be subjective. The applied force increase as well as the ratings of MMTs are based on the manual ability and ‘feeling’ of the tester. By measuring the dynamics and kinematics during the MTT, the force increase and breaking point can be objectified. The question remains whether the measured AF parameters support the subjective MMT ratings of the tester. Since the results of elbow and hip flexors showed similar characteristics, they will be considered together. All 84 MMTs at pre were assessed as ‘unstable’ by the testers. The MMTs at post and end were rated as ‘stable’ in the majority of trials (164 of 173), as ‘unstable’ in four of 173 cases and as ‘unclear’ in six of 173 cases. Regarding MMTs rated as either ‘unstable’, ‘stable’ or ‘unclear’, the ratio of AFiso_max_ to AF_max_ amounted to 50.27 ± 13.15%, 99.69 ± 0.64% or 97.95 ± 2.61%, respectively. It can be concluded that the testers’ MMT ratings were in accordance with the measured AF. Under unstable conditions, AFiso_max_ was only half as high as for the stable tests. The AF values rated as unclear in the MMTs were rather in accordance with the stable ones. Obviously, they showed a high AFiso_max_. However, during the MMT, the testers felt higher suspensions and the muscle resistance felt ‘softer’. The values of stable MMTs support the previous findings, in which the ratio of AFiso_max_ to AF_max_ was ≥99% [50,51,52]. Unstable MMTs previously revealed values of ~56%, which is slightly higher than the ~50% found here. This might be attributable to the fact that the previous studies were performed on healthy participants who were affected temporarily by unpleasant odors or imagery. Unhealthy individuals with long COVID seem to show—at least in part—even stronger impairment of muscular adaptation. Some patients showed extremely low AFiso_max_ values; the lowest was 15% for AF_max_ for hip flexors and 14% for elbow flexors. This is interpreted as a—partly extremely—impaired muscular adaptation, probably due to the long COVID state. However, it cannot be stated whether their muscular adaptation was already impaired before SARS-CoV-2 infection. Based on the findings, it can be concluded that the MMT ratings of both experienced testers were strongly in accordance with the measured AF values. Therefore, AF measured by the objectified MMT seems to be a suitable biomechanical parameter to evaluate the muscular function in adaptation to an external increasing force.

### 4.2. Adaptive Force in the Recovery Process of Long COVID

Fatigue is considered as the main symptom of long COVID [73,74,75]. The link between fatigue and muscle weakness has already been raised previously [11,15,30,76,77,78,79]. That is why maximal strength is partly investigated in post-infectious syndromes or ME/CFS. As was mentioned in the introduction, the findings have been inconclusive until now [41,42,43,44]. In the presented study, AF_max_ did not differ significantly between the three time points, as was assumed. Since AF_max_ was previously found to be similar to MVIC [45,46,47,48,49,50,51,52], the assumption that maximal forces (eccentric/MVIC) might not be suitable parameters to investigate patients in post-infectious states is supported. However, one outlier existed here, who showed extremely low AF_max_ values at pre. This could be a hint that some individuals suffering from post-infectious syndromes or ME/CFS may also have significantly reduced common maximal strengths, as was found in [43,44]. Nevertheless, the results of AF_max_ can also explain, why other investigations did not find such differences [41,42].

The findings for AFiso_max_ suggest that the holding capacity seems to be a more sensitive biomechanical parameter to assess muscle function. AFiso_max_ was significantly lower with very large effect sizes for pre vs. post and pre vs. end, whereby post vs. end did not differ significantly, according to the hypothesis. In the long COVID state (pre), the patients were not able to maintain an isometric position while trying to adapt to the increasing applied force. Muscles gave way at less than half of the maximal AF. Hence, patients could not exert their maximal strengths at this stage. This was further supported by the ratio of AFiso_max_ to AF_max_, which was significantly reduced at pre.

As the main result of the study, AFiso_max_ turned out to be a sensitive parameter for a long COVID state, because 100% of the patients showed initially clear instability (this was also the case for the other 20 patients measured in long COVID state, but who were not included in the study). To the authors’ knowledge, only one study assessed muscle strength in SARS-CoV-2 patients [80]. MVIC was measured directly at the discharge of elderly hospitalized patients. Thereby, 73% and 86% of patients showed a ‘weakness’ for biceps brachii and quadriceps femoris muscles, respectively. Muscle weakness was defined as strength which “was inferior to 80% of the predicted normal value” based on Andrews et al. [81]. However, those patients are not comparable with those included in the study, since measurements were executed at the end of acute infection following a period of hospitalization (averagely 20.7 days). More than 90% received oxygen supply and all of them were pharmacologically treated. Jäkel et al. reported a sensitivity of ~70% and ~86% for maximal hand grip strength in CFS/ME patients aged 20–39 years and 50–59 years, respectively (prior to the COVID-19 pandemic) [43].

AFiso_max_ of all long COVID patients responded immediately following the treatment at the first appointment with a clear and significant increase. This instant change leads to the assumption that AFiso_max_ does not reflect the maximal strength capacity but a functional aspect of motor control that can be influenced by stimuli. It can switch immediately from instability to stability or vice versa. This was shown in previous studies involving healthy participants [50,51,52]. The health condition of the long COVID patients in this study was not improved directly after the first treatment (except for one patient), but the motor control already clearly responded. It is hypothesized that the motor reaction could have been a first hint at a helpful intervention, at least in a share of subjects. The actual causality remains unclear. There could have been helpful treatment methods, but also possible mental factors, such as an empathetic atmosphere or the like.

The significant differences between pre and end revealed that the holding capacity was not only substantially improved, but even fully normalized until recovery. This result has to be discussed independently of the possible causations of the improvement. Because the study was non-clinical, no control group was included. Therefore, the reasons for improvement of health conditions and AF parameters remain unclear.

Considering the queried symptoms, it was visible that they behave inversely proportional to the holding capacity: at pre, the symptom intensity was significantly higher in most items compared to timepoint end (*p* < 0.001 to 0.031; except for fever (*p* = 0.281) and loss of smell/taste (*p* = 0.062)), whereby AFiso_max_ and the ratio of AFiso_max_ to AF_max_ was significantly lower at pre vs. end with large effect sizes of > 2.42. This indicates an inverse correlation of the health condition and holding capacity. The directionality and causation of this connection can only be assumed. Since the holding capacity was improved already directly after the first treatment (post), the holding capacity cannot be a direct indicator for the improvement in health. Moreover, it remains unclear whether that observed instant improvement was sustainable. It seems to be likely that the motor response was a more transitional phenomenon at the beginning. MMTs at following treatment appointments showed a fallback to muscular instability for the most patients; however, this was not verified by objective measures. Because the output-measurements (end) were not carried out after an immediately preceding treatment, the observed stability could be interpreted as a part of the improved health state. We assume that the holding capacity is regained prior to the decrease of symptom intensity. Hence, after suitable treatments, the functionality is first restored. A probable improvement in the health condition is time-delayed and might possibly depend on the sustainability of this regained functionality, mirrored by the stable muscle function.

### 4.3. Neurophysiological Considerations with Respect to the Reaction of AF in Long COVID

The discussion on the etiology of long COVID should not be opened here in detail. Brain stem dysfunction [36], a reduced cerebral blood flow [31,82] and the involvement of the ANS [25,26,30,31,37,38,39] were discussed. Recently, preinfection psychological distress was reported as a risk factor for long COVID [75,83]. This is in line with the self-reported stress prior to acute infection regarding the patients in the present study. Central structures, such as the brain stem, thalamus, basal ganglia, cerebellum, inferior olivary nucleus, cingulate cortex and more are involved in processing and controlling nociception, emotions and motor control [84,85,86,87,88]. Hence, the influence of possibly interfering inputs in the complex control circuitries of motor function are conceivable. The adaptive holding capacity in reaction to an external increasing force was suggested to be especially vulnerable regarding such stimuli. The length-tension control with respect to an increasing external load challenges the regulation and control processes of motor control in a specific way (for detailed discussion see [49,50,51,52]). Therefore, it is conceivable that a health state, such as long COVID, can influence the holding capacity. Based on the findings of previous studies on the influence of emotions on AF in healthy participants [50,51,52] and on long-term practical experience that mental stress can reduce the holding capacity, we assume that the motor output in the sense of AF could have been impaired already prior to SARS-CoV-2 infection because of mental stress. This might have affected the functionality of the human system on different levels. Especially an impairment of the immune system is known to be associated with mental distress [83,89,90,91,92,93,94]. Hence, the individually perceived mental stress could have diminished the resilience of the individual with regard to the virus and, probably, could have impeded the recovery of the acute infection, resulting in long COVID. Wang et al. [83] highlighted that the findings that psychological distress is a risk factor for long COVID “should not be misinterpreted as supporting a hypothesis that post-COVID-19 conditions are psychosomatic”. We concur with this statement. From our point of view, mental stress might lead to disbalances of different bodily systems, e.g., the immune system [83,89,90] or the ANS [95]. This, in turn, could lower the resilience and might favor long COVID. We interpret the long COVID state rather as a sign of dysfunction. The found instability of the holding muscle function might be a part of the complex physiological functional disbalance in long COVID patients.

The onset of oscillation (AFosc) might also reflect an impaired functionality. The neuromuscular system is known to be characterized by oscillations. AFosc was significantly higher for pre vs. post and pre vs. end, as was hypothesized. Moreover, in all of the 84 MMTs of elbow and hip flexors at pre (rated as unstable), oscillations arose—if at all—after the breaking point, thus during muscle lengthening. For the remaining 173 MMTs at post and end (mostly rated as stable), the up-swing of oscillations arose regularly during isometric actions. Those findings support the previous ones that, in case of stability, oscillations occur during isometric muscle action; in case of instability, they do not arise. This indicates that oscillations might be a prerequisite for the stable adaptation in the sense of AF, as was suggested previously [50,51,52]. The evidence consolidates that oscillations are playing a major role in the neuromuscular adaptation with respect to external forces.

Based on the connection of physiological disbalances and motor control, the AF might be a suitable biomechanical parameter to check for such functional impairments. Due to the immediate response of the holding capacity to supporting or disrupting inputs, the recovery process of long COVID could also be controlled, and a potentially supportive therapy approach might be ascertained by assessing the holding capacity.

### 4.4. Limitations

One limitation was the non-standardized duration from post to end measurements. Due to the individual recovery process, this limitation is difficult to resolve. The duration depended on the self-reported health state of the patients. This self-report is another limitation. Further studies could include a more quantitative assessment of the health state. However, the individual feeling of health is the most important one, also for return to work. Furthermore, the study was not blinded. The testers were aware of the patients’ health state. However, the evaluation of the slope and AF_max_ revealed statistically similar values between the three time points. Only AFiso_max_, as well as AFosc, showed significant differences between pre vs. post and pre vs. end. This strongly indicates that the AF assessment was not influenced by lack of blinding.

## 5. Conclusions

The investigation of the AF in patients with long COVID and in the course of their recovery process revealed that the holding capability was significantly reduced in long COVID state and was stabilized after the first treatment and with substantial health improvement. AF_max_ did not reflect this difference. The holding capacity seems to be sensitive but is assumed to be not specific for long COVID. Nonetheless, its assessment might support the diagnostics of long COVID and especially the choice of the individual helpful therapy approach, since the holding function can switch immediately from instability to stability. This should be used to identify a treatment tailored to the patient’s individual conditions and requirements. It is concluded that the assessment of AFiso_max_ could be a supportive biomechanical parameter to assess the functional health state, follow up and recovery process in patients with long COVID. The next step should be to investigate the mentioned treatment approaches in a clinical design. Based on the present study, it cannot be judged whether the treatments were the reason for the recovery. Possibly, other received treatments or a spontaneous recovery over time could have led to the improved health state. In case the treatment approaches are verified positively, this would be a big step towards diagnostics and therapy with regard to long COVID. This would have major socioeconomic implications.

## Figures and Tables

**Table 1:** Subjective ratings of the manual muscle tests by the testers. The number of MMTs assessed as unstable, stable or as unclear for hip and elbow flexors for each time point is given.

	Hip Flexors (n = 144)			Elbow Flexors (n = 118)		
MMT Rating	pre	Post	End	pre	Post	End
unstable	48	2	0	39	2	1
stable	0	42	47	0	35	40
unclear	0	3	2	0	1	0

**Table 2:** Parameters of the Adaptive Force (AF) of elbow and hip flexors. Arithmetic means ± standard deviations (M ± SD), lower and upper borders of 95% confidence intervals (CIs) and values of the RM ANOVA (F-statistics, degrees of freedom (df), significance *p* and effect size η^2^) or of the Friedman test (z, df, *p*, effect size Kendall’s W) are given for all AF parameters at each time point (pre: in long COVID state–before treatment, post: directly after treatment, end: recovery) of elbow (n = 14) and hip flexors (n = 17). Parameters: maximal AF (AF_max_) (N), maximal isometric AF (AFiso_max_) (N), ratio AFiso_max_ to AF_max_ (%), AF at onset of oscillations (AFosc) (N) and the ratios of AFosc to AF_max_ (%) and AFosc to AFiso_max_ (%), as well as the slope of force rise (lg(N/s)).

Parameter	Time Point	M ± SD	95%-CI	F (df_1_,df_2_) or z (df)	Significance *p*	η^2^/Kendall’s W
elbow flexors (n = 14)						
AF_max_ (N)	pre	177.02 ± 53.47	149.01; 205.03	1.054 (1.43,18.63) ^a^	0.345	-
	post	184.74 ± 39.02	164.27; 205.15			
	end	187.87 ± 52.00	160.63; 215.11			
AFiso_max_ (N)	pre	87.92 ± 54.41	59.42; 116.42	114.772 (2,26)	**<0.0001**	0.898
	post	182.26 ± 38.58	162.05; 202.47			
	end	187.22 ± 52.15	159.90; 214.53			
Ratio AFiso_max_ to AF_max_ (%)	pre	46.58 ± 15.91	38.25; 54.91	25.064 (2) ^b^	**<0.0001**	0.895 ^b^
	post	98.73 ± 3.01	97.15; 100.31			
	end	99.62 ± 0.96	99.12; 100.13			
AFosc (N)	pre	170.95 ± 49.17	145.20; 196.71	5.274 (2,26)	**0.012**	0.289
	post	144.54 ± 44.83	121.06; 168.03			
	end	146.51 ± 48.64	121.04; 171.99			
Ratio AFosc to AF_max_ (%)	pre	96.87 ± 2.85	95.38; 98.36	23.403 (2,26)	**<0.0001**	0.643
	post	76.95 ± 11.89	70.73; 83.18			
	end	76.98 ± 11.09	71.17; 82.79			
Ratio AFosc to AFiso_max_ (%)	pre	258.83 ± 110.74	200.82; 316.84	34.701 (1.02,13.19) ^a^	**<0.0001**	0.727
	post	78.06 ± 12.30	71.62; 84.50			
	end	77.28 ± 10.92	71.55; 83.00			
Slope lg(N/s)	pre	1.85 ± 0.23	1.73; 1.98	1.282 (2,26)	0.294	-
	post	1.87 ± 0.18	1.78; 1.97			
	end	1.90 ± 0.21	1.79; 2.01			
hip flexors (n = 17)						
AF_max_ (N)	pre	174.98 ± 50.03	148.77; 148.77	0.015 (2,32) ^a^	0.952	-
	post	175.67 ± 40.95	154.22; 197.12			
	end	174.21 ± 46.78	149.71; 198.72			
AFiso_max_ (N)	pre	88.30 ± 40.67	66.99; 109.61	88.739 (1.47,23.45) ^a^	**<0.0001**	0.847
	post	173.30 ± 41.75	151.43; 195.18			
	end	174.06 ± 46.80	149.54; 198.58			
Ratio AFiso_max_ to AF_max_ (%)	pre	49.25 ± 12.01	42.96; 55.54	32.109 (2) ^b^	**<0.0001**	0.944 ^b^
	post	98.54 ± 3.44	96.74; 100.35			
	end	99.91 ± 0.39	99.70; 100.11			
AFosc (N)	pre	167.10 ± 43.80	144.16; 190.04	27.952 (2,32)	**<0.0001**	0.636
	post	116.47 ± 39.63	95.71; 137.23			
	end	110.06 ± 40.81	88.68; 131.44			
Ratio AFosc to AF_max_ (%)	pre	95.19 ± 5.59	92.26; 98.12	53.417 (2,32)	**<0.0001**	0.77
	post	65.62 ± 11.56	59.57; 71.68			
	end	62.01 ± 13.74	54.81; 69.21			
Ratio AFosc to AFiso_max_ (%)	pre	223.06 ± 69.65	186.57; 259.54	78.199 (1.07,17.11) ^a^	**<0.0001**	0.83
	post	66.88 ± 13.18	59.97; 73.78			
	end	62.07 ± 13.78	54.85; 69.29			
Slope lg(N/s)	pre	1.85 ± 0.18	1.75; 1.94	3.260 (1.45,21.73) ^a^	0.071	-
	post	1.93 ± 0.15	1.85; 2.00			
	end	1.89 ± 0.14	1.81; 1.97			

^a^ Greenhouse–Geisser correction was applied for the RM ANOVA. ^b^ Friedman test was performed with effect size Kendall’s W. Significant results are displayed in bold.

**Table 3:** Stress level and symptom intensity. Given are the arithmetic means, standard deviations (M ± SD) and range including the number of patients (n) for job-related and personal stress level, as well as for the intensity of the queried common long COVID symptoms (from 0 = no, 10 = very strong) for the different time points before COVID infection (retrospectively), during long COVID state (corresponds to pre) and at recovery/substantial heath improvement (end). The values of the Friedman test comparing the three time points, significance *p* and effect size Kendall’s W are given. The results of the pairwise comparisons are indicated in superscript.

Stress M ± SD (Range, n)	Before COVID	Long COVID State (pre)	End	Friedman Test	Significance *p*	Effect Size Kendall’s W
Stress level job-related	4.23 ± 2.56 (0–9, n = 11)	5.64 ± 2.95 (0–10, n = 14 *)	2.29 ± 3.17 (0–8, n = 12)	0.667	0.717	-
Stress level personal life	3.77 ± 2.70 (2–10, n = 12)	4.76 ± 2.75 (0–10, n = 17)	3.29 ± 3.53 (0–9, n = 12)	4.056	0.132	-
**Symptoms** **M ± SD** **(range)**	**n = 14**	**n = 14**	**n = 13**			
Depression/anxiety	1.43 ± 2.21 (0–8)	3.96 ± 3.78 (0–10)	1.58 ± 2.33 (0–7)	9.389	**0.009 ^1,2^**	0.361
Fatigue	0.43 ± 0.76 (0–2)	7.75 ± 2.50 (1–10)	2.23 ± 2.67 (0–7.5)	22.217	**<0.001 ^1,2^**	0.855
Post-exertion malaise	0.57 ± 1.40 (0–5)	8.14 ± 1.96 (3–10)	2.23 ± 3.06 (0–9)	20.311	**<0.001 ^1,2^**	0.781
Muscle pain	0.29 ± 0.61 (0–2)	5.64 ± 4.27 (0–10)	1.65 ± 2.81 (0–8)	14.800	**0.001 ^1,2^**	0.569
Chest pain/tightness	0.00 ± 0.00	3.43 ± 3.01 (0–9.5)	0.88 ± 1.23 (0–4)	18.667	**<0.001 ^1,2^**	0.718
Breathing difficulties	0.29 ± 0.61 (0–2)	4.29 ± 2.37 (1–8)	1.23 ± 1.36 (0–3)	23.106	**<0.001 ^1,2^**	0.889
Cough	0.14 ± 0.36 (0–1)	2.23 ± 3.00 (0–10)	0.46 ± 0.97 (0–3)	16.267	**<0.001 ^1,2^**	0.678
Strong/fast heartbeat	0.36 ± 1.08 (0–4)	4.93 ± 4.03 (0–10)	0.69 ± 1.18 (0–3)	17.882	**<0.001 ^1,2^**	0.688
Concentration/memory problems	0.43 ± 0.76 (0–2)	5.96 ± 3.20 (0–10)	2.88 ± 2.60 (0–9)	23.130	**<0.001 ^1,2^**	0.890
Dizziness	0.14 ± 0.36 (0–1)	4.71 ± 3.81 (0–10)	0.92 ± 1.98 (0–7)	16.800	**<0.001 ^1,2^**	0.646
Headache	0.64 ± 1.28 (0–4)	5.29 ± 4.07 (0–10)	0.81 ± 1.28 (0–3)	18.242	**<0.001 ^1,2^**	0.702
Loss of smell or taste	0.00 ± 0.00	4.39 ± 4.85 (0–10)	1.35 ± 3.16 (0–10)	13.923	**0.001 ^1^**	0.536
Fever	0.00 ± 0.00	2.14 ± 3.66 (0–10)	0.23 ± 0.83 (0–3)	7.538	**0.023**	0.290

* Three patients made no statement because of sick leave. ^1^ The pairwise comparison of ‘before COVID’ vs. ‘long COVID state’ was significant: *p* < 0.05. ^2^ The pairwise comparison of ‘long COVID state’ vs. end was significant: *p* < 0.05.

## Data Availability

The data presented in this study are available in the article and Supplementary Materials.

## Supplementary Materials

The following supporting information can be downloaded at: https://www.mdpi.com/article/10.3390/diagnostics13050882/s1, Table S1: Patient characteristics, COVID specific durations and number of treatments; Table S2: Intensity of symptoms; Table S3: Ratings of the manual muscle tests by the tester; Table S4: Maximal adaptive force of elbow flexors; Table S5: Maximal isometric adaptive force of elbow flexors; Table S6: Adaptive force at onset of oscillations of elbow flexors; Table S7: Slope of force increase for elbow flexors; Table S8: Maximal adaptive force of hip flexors; Table S9: Maximal isometric adaptive force of hip flexors; Table S10: Adaptive force at onset of oscillations of hip flexors; Table S11: Slope of force increase for hip flexors.
