# Supplementary material for: The Adaptive Force as a Potential Biomechanical Parameter in the Recovery Process of Patients with Long COVID
Source: Diagnostics (Basel). 2023 Feb 25;13(5):882. doi: 10.3390/diagnostics13050882 (PMC10000769; doi:10.3390/diagnostics13050882)
Supplement: Supplementary file 1 [file diagnostics-13-00882-s001.zip › diagnostics-2075572-supplementary.pdf]

diagnostics

Supplementary material

Article

The Adaptive Force as potential biomechanical parameter in the recovery process of patients with long COVID

(Schaefer LV, Bittmann FN)

Content

Table S1. Patients characteristics, COVID specific durations and number of treatments.....................2

Table S2. Intensity of symptoms 3

Table S3. Ratings of manual muscle tests by the tester..4

Table S4. Maximal Adaptive Force of elbow flexors...5

Table S5. Maximal isometric Adaptive Force of elbow flexors...6

Table S6. Adaptive Force at onset of oscillations of elbow flexors..7

Table S7. Slope of force increase for elbow flexors...8

Table S8. Maximal Adaptive Force of hip flexors..9

Table S9. Maximal isometric Adaptive Force of hip flexors..10

Table S10. Adaptive Force at onset of oscillations of hip flexors 11

Table S11. Slope of force increase for hip flexors..11

Table S1. Patients characteristics, COVID specific durations and number of treatments

Gender, age and anthropometric data of patients, duration (days) from start of COVID to pre measurements and from pre to end measurements as well as number of treatment from pre to end measurements in the practice.

					duration (days)		
ID	gender (female = 1, male = 2)	age (years)	body height (cm)	body mass (kg)	start COVID to pre	pre to end	number of treatments from pre to end
1	1	63	172	61	176	37	2
2	1	51	167	67	200	89	5
3	1	24	168	65	240	31	1
4	1	59	163	63	278	166	4
5	2	43	185	95	420	39	4
6	1	25	170	56	205	145	3
7	1	53	167	82	325	111	5
8	1	52	173	65	677	56	7
9	1	21	172	66	409	68	4
10	1	50	158	62	458	28	2
11	1	61	176	93	383	49	3
12	1	42	174	103	47	114	1
13	1	43	170	63	688	77	5
14	1	25	160	57	34	27	1
15	2	58	190	94.5	48	111	3
16	2	46	-	-	32	34	5
17	1	53	170	76	53	26	1

Table S2. Intensity of symptoms. *(before = retrospectively for before COVID infection; LC = Long COVID (pre trials), end = end measurements)*

ID	job-related stress			personal stress			depression/ anxiety			fatigue			post-exertion malaise			muscle pain			chest pain/ tightness		
	before	LC	end	before	LC	end	before	LC	end	before	LC	end	before	LC	end	before	LC	end	before	LC	end
1	0	0	0	2	4	1	1	4	1	1	6	0	0	3	0	0	0	0	0	0	0
2	3	7	1	2	7	1	0	5	2	0	10	0	0	7	0	2	10	1	0	5	1.5
3	-	4	-	-	6	-	3	10	0	0	9	0	0	10	0	0	9	0	0	3	0
4	3	10	7	10	10	7	2	10	7	0	10	0	0	10	0	0	10	0	0	9.5	4
5	-	5	-	-	1	-	-	-	-	-	-	-	-	-	-	-	-	-	-	-	-
6	5	5	6.5	4	4	8.5	0	0	1.5	2	8	3.5	0	8	0	1	10	3.5	0	0	0
7	2.5	8	8	2.5	8	7	2	7.5	2	2	8.5	7.5	2	9	7	1	9	7	0	5	2
8	4	-	0	3	2	1	3	2	0	0	10	2	0	10	2	0	8	2	0	8	1
9	7	5	4	8	8	9	8	9	6	0	9	6	0	10	5	0	8	0	0	4	2
10	-	9	0	-	7	3	0	0	0	0	9	6	0	8	9	0	5	8	0	4	1
11	3	-	-	3	3	-	1	2	-	0	8	-	1	8	-	0	0	-	0	0	-
12	-	5	0	-	2	0	0	0	0	0	5	0	0	6	0	0	8	0	0	4.5	0
13	3	6	0	3	6	2	0	4	1	0	6	2	5	9	3	0	0	0	0	1	0
14	9	0	0	2	0	0	0	0	0	0	1	1	0	7	3	0	0	0	0	4	0
15	7	-	1	2	5	0	0	2	0	1	9	1	0	9	0	0	2	0	0	0	0
16	-	8	-	-	5	-	-	-	-	-	-	-	-	-	-	-	-	-	-	-	-
17	-	7	-	-	3	-	-	-	-	-	-	-	-	-	-	-	-	-	-	-	-

ID	breathing difficulties			cough			strong/fast heartbeat			concentration/memory problems			dizziness			headache			loss of taste/smell			fever		
	before	LC	end	before	LC	end	before	LC	end	before	LC	end	before	LC	end	before	LC	end	before	LC	end	before	LC	end
1	0	2	0	1	2	0	0	0	0	0	0	0	0	0	0	0	0	0	0	10	1	0	0	0
2	0	7	3	0	10	2	0	0	0	0	8	3	0	7	0	1	10	2	0	10	0	0	0	0
3	0	1	0	0	2	0	0	4	0	0	10	2	0	9	0	0	10	0	0	9	0	0	0	0
4	0	8	3	0	1	0	0	10	3	0	8	4	0	8	0	0	10	0	0	10	0	0	0	0
5	-	-	-	-	-	-	-	-	-	-	-	-	-	-	-	-	-	-	-	-	-	-	-	-
6	0	4	0	0	0	0	0	5	0	0	7	2.5	0	0	0	4	10	2.5	0	0	0	0	0	0
7	1	3	2	1	3	1	1	8	3	2	8.5	5	0	8	2	3	6	3	0	9.5	6.5	0	8	3
8	0	5	1	0	1	0	0	6	1	0	1	0	0	0	0	0	3	0	0	0	0	0	0	0
9	2	6	0	0	0	0	0	8	0	0	8	6	0	8	2	0	8	0	0	10	10	0	7	0
10	0	8	3	0	0	0	4	8	2	0	10	9	0	10	7	0	8	3	0	1	0	0	0	0
11	1	4	-	0	0	-	0	0	-	1	7	-	1	3	-	1	2	-	0	0	-	0	0	-
12	0	5	0	0	2	0	0	10	0	0	3	1	0	7	0	0	5	0	0	0	0	0	10	0
13	0	2	1	0	0	0	0	1	0	2	5	3	1	4	1	0	0	0	0	0	0	0	0	0
14	0	4	3	0	7	3	0	0	0	1	3	1	0	0	0	0	0	0	0	0	0	0	0	0
15	0	1	0	0	1	0	0	9	0	0	5	1	0	2	0	0	2	0	0	2	0	0	5	0
16	-	-	-	-	-	-	-	-	-	-	-	-	-	-	-	-	-	-	-	-	-	-	-	-
17	-	-	-	-	-	-	-	-	-	-	-	-	-	-	-	-	-	-	-	-	-	-	-	-

Table S3. Ratings of manual muscle tests by the tester.

Ratings of each MMT of elbow and hip flexors are given for each time point (pre = in Long COVID state prior to first treatment; post = immediatley after first treatment; end = at end measurements); 0 = unstable, 1 = stable, 2 = unclear.

	**elbow**									**hip**									
	**pre**			**post**			**end**			**pre**			**post**			**end**			
ID	MMT 1	MMT 2	MMT 3	MMT 1	MMT 2	MMT 3	MMT 1	MMT 2	MMT 3	MMT 1	MMT 2	MMT 3	MMT 1	MMT 2	MMT 3	MMT 1	MMT 2	MMT3	MMT4
1	0	0	-	1	1	-	1	1	-	0	0	-	1	1	-	1	1	-	-
2	0	0	0	1	1	-	1	1	1	0	0	0	1	1	-	1	1	1	-
3	0	0	0	2	1	1	1	1	1	0	0	0	1	1	1	1	1	1	-
4	0	0	0	1	1	1	1	1	1	0	0	0	1	1	1	1	1	1	-
5	-	-	-	-	-	-	-	-	-	0	0	0	1	1	1	1	1	1	-
6	0	0	0	1	1	1	1	1	1	0	0	0	1	1	1	1	1	1	-
7	0	0	0	1	1	1	1	1	1	0	0	0	1	1	1	1	1	1	-
8	0	0	0	1	1	1	1	1	1	0	0	0	1	1	1	1	1	1	-
9	0	0	0	1	1	1	1	1	1	0	0	0	1	1	1	1	1	1	-
10	0	0	0	1	1	1	1	1	1	0	0	0	2	2	2	2	1	1	-
11	0	0	-	1	1	-	1	1	1	0	0	-	1	1	-	2	-	-	-
12	0	0	0	1	1	1	1	1	1	0	0	0	1	1	1	1	1	1	1
13	0	0	-	0	0	-	1	1	1	0	0	-	0	0	-	1	1	1	-
14	0	0	0	1	1	1	0	1	1	0	0	0	1	1	1	1	1	1	-
15	0	0	0	1	1	1	1	1	1	0	0	0	1	1	1	1	1	1	-
16	-	-	-	-	-	-	-	-	-	0	0	0	1	1	1	1	1	1	-
17	-	-	-	-	-	-	-	-	-	0	0	0	1	1	1	1	1	1	-

Red colored numbers indicate that data of handheld device was not recorded due to technical problems.

Table S4. Maximal Adaptive Force of elbow flexors.

Values of maximal Adaptive Force (AF_{max}) in N for all measurements (M1, M2, M3) of each patient (ID) for each timepoint (pre = Long COVID state prior to first treatment, post = immediately after first treatment, end = at end measurements). Arithmetic mean (M), standard deviation (SD) and coefficient of variation (CV) for the trials of each patient at each timepoint are given.

	**pre**						**post**						**end**					
ID	M1	M2	M3	M	SD	CV	M1	M2	M3	M	SD	CV	M1	M2	M3	M	SD	CV
1	201.029	191.731	-	196.380	6.575	0.033	184.956	187.040	-	185.998	1.474	0.008	205.717	188.920	-	197.319	11.877	0.060
2	157.588	153.952	168.398	159.979	7.514	0.047	203.701	209.455	-	206.578	4.069	0.020	189.391	196.672	205.487	197.183	8.060	0.041
3	161.449	142.639	177.157	160.415	17.282	0.108	176.288	196.231	181.615	184.711	10.326	0.056	202.338	192.393	192.434	195.722	5.730	0.029
4	199.522	205.798	203.861	203.060	3.214	0.016	208.672	202.927	217.163	209.587	7.162	0.034	161.358	205.233	199.963	188.851	23.955	0.127
5	-	-	-	-	-	-	-	-	-	-	-	-	-	-	-	-	-	-
6	148.762	144.192	168.551	153.835	12.948	0.084	132.387	154.191	150.036	145.538	11.577	0.080	145.287	154.942	164.290	154.840	9.502	0.061
7	163.993	155.935	159.445	159.791	4.040	0.025	177.310	193.954	198.025	189.763	10.975	0.058	199.822	190.819	193.897	194.846	4.576	0.023
8	*	*	170.833	170.833	-	-	165.433	179.260	169.695	171.463	7.081	0.041	176.938	185.108	180.410	180.819	4.100	0.023
9	55.898	63.347	65.035	61.427	4.862	0.079	164.707	121.943	152.671	146.440	22.052	0.151	115.120	132.725	159.739	135.861	22.474	0.165
10	193.884	167.308	178.792	179.995	13.329	0.074	137.531	144.191	132.191	137.971	6.012	0.044	125.467	139.058	142.795	135.773	9.119	0.067
11	148.751	142.914	-	145.833	4.127	0.028	135.718	125.988	-	130.853	6.880	0.053	87.870	113.572	110.106	103.849	13.947	0.134
12	268.935	242.743	*	255.839	18.521	0.072	266.977	259.999	255.490	260.822	5.788	0.022	284.197	297.660	300.614	294.157	8.751	0.030
13	180.080	161.929	-	171.005	12.835	0.075	182.736	182.698	-	182.717	0.027	0.000	172.173	182.103	186.017	180.098	7.137	0.040
14	153.265	156.100	185.214	164.860	17.684	0.107	179.014	184.283	179.662	180.986	2.873	0.016	176.220	173.647	201.165	183.677	15.199	0.083
15	311.661	285.867	287.622	295.050	14.412	0.049	262.945	248.218	246.312	252.492	9.103	0.036	281.579	292.957	287.067	287.201	5.690	0.020
16	-	-	-	-	-	-	-	-	-	-	-	-	-	-	-	-	-	-
17	-	-	-	-	-	-	-	-	-	-	-	-	-	-	-	-	-	-

- = not measured

* = not recorded because of technical problems

Table S5. Maximal isometric Adaptive Force of elbow flexors.

Values of maximal isometric Adaptive Force ($AFiso_{max}$) in N for all measurements (M1, M2, M3) of each patient (ID) for each timepoint (pre = Long COVID state prior to first treatment, post = immediately after first treatment, end = at end measurements). Arithmetic mean (M), standard deviation (SD) and coefficient of variation (CV) for the trials of each patient at each timepoint are given.

	**pre**						**post**						**end**					
ID	M1	M2	M3	M	SD	CV	M1	M2	M3	M	SD	CV	M1	M2	M3	M	SD	CV
1	116.840	131.960	-	124.400	10.691	0.086	184.956	187.040	-	185.998	1.474	0.008	205.717	188.920	-	197.319	11.877	0.060
2	83.778	56.030	66.777	68.862	13.991	0.203	203.701	208.850	-	206.276	3.641	0.018	189.391	196.672	205.487	197.183	8.060	0.041
3	111.447	28.500	73.504	71.150	41.524	0.584	174.240	196.231	181.615	184.029	11.192	0.061	202.338	192.393	192.434	195.722	5.730	0.029
4	84.033	132.316	74.399	96.916	31.033	0.320	208.672	202.927	217.163	209.587	7.162	0.034	161.358	205.233	199.963	188.851	23.955	0.127
5	-	-	-	-	-	-	-	-	-	-	-	-	-	-	-	-	-	-
6	60.494	71.115	55.031	62.213	8.179	0.131	132.387	154.191	150.036	145.538	11.577	0.080	145.287	143.659	164.290	151.079	11.470	0.076
7	101.435	110.573	118.205	110.071	8.396	0.076	177.310	193.954	198.025	189.763	10.975	0.058	199.822	174.592	193.897	189.437	13.193	0.070
8	*	*	68.553	68.553	-	-	165.433	179.260	169.695	171.463	7.081	0.041	176.938	185.108	180.410	180.819	4.100	0.023
9	16.079	18.900	26.689	20.556	5.495	0.267	164.707	121.943	152.671	146.440	22.052	0.151	115.120	132.725	159.739	135.861	22.474	0.165
10	147.134	64.692	39.637	83.821	56.244	0.671	137.531	144.191	132.191	137.971	6.012	0.044	125.467	139.058	142.795	135.773	9.119	0.067
11	59.188	19.329	-	39.259	28.185	0.718	135.718	125.988	-	130.853	6.880	0.053	87.870	113.572	110.106	103.849	13.947	0.134
12	168.473	144.127	*	156.300	17.215	0.110	266.977	259.999	236.573	254.516	15.926	0.063	284.197	297.660	300.614	294.157	8.751	0.030
13	50.372	29.914	-	40.143	14.466	0.360	161.224	164.263	-	162.744	2.149	0.013	172.173	182.103	186.017	180.098	7.137	0.040
14	85.105	59.351	31.536	58.664	26.791	0.457	158.167	184.283	179.662	174.037	13.937	0.080	176.220	173.647	201.165	183.677	15.199	0.083
15	243.722	225.108	220.990	229.940	12.112	0.053	262.945	248.218	246.312	252.492	9.103	0.036	281.579	292.957	287.067	287.201	5.690	0.020
16	-	-	-	-	-	-	-	-	-	-	-	-	-	-	-	-	-	-
17	-	-	-	-	-	-	-	-	-	-	-	-	-	-	-	-	-	-

- = not measured

* = not recorded because of technical problems

Table S6. Adaptive Force at onset of oscillations of elbow flexors.

Values of Adaptive Force at onset of oscillations (AFosc) in N for all measurements (M1, M2, M3) of each patient (ID) for each timepoint (pre = Long COVID state prior to first treatment, post = immediately after first treatment, end = at end measurements). Arithmetic mean (M), standard deviation (SD) and coefficient of variation (CV) for the trials of each patient at each timepoint are given.

	pre						post						end					
ID	M1	M2	M3	M	SD	CV	M1	M2	M3	M	SD	CV	M1	M2	M3	M	SD	CV
1	195.182	186.454	-	190.818	6.172	0.032	161.917	158.175	-	160.046	2.646	0.017	167.050	161.346	-	164.198	4.033	0.025
2	143.128	x	155.050	149.089	8.430	0.057	147.138	186.532	-	166.835	27.856	0.167	143.533	150.063	186.913	160.170	23.389	0.146
3	159.744	142.639	141.460	147.948	10.233	0.069	160.652	171.586	152.782	161.673	9.444	0.058	175.665	162.153	188.168	175.329	13.011	0.074
4	190.696	205.798	201.816	199.437	7.827	0.039	177.803	192.623	212.519	194.315	17.420	0.090	138.579	149.278	184.305	157.387	23.917	0.152
5	-	-	-	-	-	-	-	-	-	-	-	-	-	-	-	-	-	-
6	148.762	144.192	168.551	153.835	12.948	0.084	72.875	64.998	51.063	62.979	11.045	0.175	105.368	119.219	99.660	108.082	10.058	0.093
7	160.531	150.327	159.445	156.768	5.604	0.036	166.057	153.753	143.329	154.380	11.377	0.074	156.776	131.027	126.026	137.943	16.500	0.120
8	*	*	162.829	162.829	-	-	142.174	142.100	126.266	136.847	9.163	0.067	159.272	162.301	163.301	161.625	2.098	0.013
9	55.898	63.347	63.261	60.835	4.276	0.070	126.609	85.034	91.180	100.941	22.441	0.222	104.387	87.542	110.784	100.904	12.006	0.119
10	188.962	167.308	178.792	178.354	10.834	0.061	90.178	82.010	132.191	101.460	26.926	0.265	88.198	117.825	107.771	104.598	15.066	0.144
11	148.751	142.631	-	145.691	4.327	0.030	87.849	84.447	-	86.148	2.406	0.028	55.886	56.855	43.266	52.002	7.581	0.146
12	251.817	236.893	*	244.355	10.553	0.043	217.136	233.795	202.150	217.694	15.830	0.073	228.405	209.043	273.077	236.842	32.840	0.139
13	177.872	160.577	-	169.225	12.229	0.072	154.103	128.940	-	141.522	17.793	0.126	161.786	147.112	172.651	160.516	12.817	0.080
14	152.517	146.579	185.214	161.437	20.805	0.129	135.019	131.441	145.565	137.342	7.343	0.053	117.460	86.381	133.958	112.600	24.158	0.215
15	281.080	274.991	262.017	272.696	9.737	0.036	255.062	176.715	172.449	201.409	46.514	0.231	179.842	206.453	270.601	218.965	46.655	0.213
16	-	-	-	-	-	-	-	-	-	-	-	-	-	-	-	-	-	-
17	-	-	-	-	-	-	-	-	-	-	-	-	-	-	-	-	-	-

- = not measured
* = not recorded because of technical problems
x = not determinable because of peculiarities of curve shape

Table S7. Slope of force increase for elbow flexors.

Logarithmic slope of force increase during AF measurement in lg(N/s)) for all measurements (M1, M2, M3) of each patient (ID) for each timepoint (pre = Long COVID state prior to first treatment, post = immediately after first treatment, end = at end measurements). Arithmetic mean (M), standard deviation (SD) and coefficient of variation (CV) for the trials of each patient at each timepoint are given.

	**pre**						**post**						**end**					
ID	M1	M2	M3	M	SD	CV	M1	M2	M3	M	SD	CV	M1	M2	M3	M	SD	CV
1	2.013	2.046	-	2.030	0.024	0.012	1.886	2.014	-	1.950	0.090	0.046	2.261	1.802	-	2.031	0.324	0.160
2	1.908	1.841	1.663	1.804	0.127	0.070	1.806	1.979	-	1.893	0.122	0.065	1.838	1.781	1.853	1.824	0.038	0.021
3	1.894	1.682	2.014	1.863	0.168	0.090	1.959	1.852	1.951	1.921	0.059	0.031	1.903	1.944	1.994	1.947	0.046	0.024
4	1.919	2.028	1.927	1.958	0.061	0.031	2.018	1.771	2.024	1.938	0.145	0.075	1.979	1.918	2.091	1.996	0.088	0.044
5	-	-	-	-	-	-	-	-	-	-	-	-	-	-	-	-	-	-
6	1.818	1.736	1.738	1.764	0.046	0.026	1.691	1.754	1.589	1.678	0.083	0.049	1.864	1.946	1.946	1.919	0.047	0.025
7	2.019	1.964	1.984	1.989	0.028	0.014	1.991	2.016	2.096	2.034	0.055	0.027	2.085	1.947	2.119	2.050	0.091	0.044
8	*	*	1.824	1.824	-	-	1.732	2.017	1.939	1.896	0.147	0.078	2.063	2.117	1.887	2.022	0.120	0.059
9	1.34221	1.29622	1.31197	1.317	0.023	0.018	1.672	1.599	1.561	1.611	0.057	0.035	1.535	1.547	1.444	1.509	0.056	0.037
10	1.924	1.97294	1.93126	1.943	0.026	0.014	1.786	1.841	1.896	1.841	0.055	0.030	1.843	1.821	x	1.832	0.015	0.008
11	1.812	1.682	-	1.747	0.092	0.053	1.676	1.629	-	1.652	0.033	0.020	1.393	1.569	1.570	1.510	0.102	0.068
12	2.255	2.185	*	2.220	0.050	0.022	2.289	2.301	2.216	2.269	0.046	0.020	2.249	2.125	2.380	2.251	0.128	0.057
13	1.543	1.486	-	1.514	0.040	0.026	1.605	1.744	-	1.675	0.099	0.059	1.863	1.864	1.703	1.810	0.092	0.051
14	1.881	1.805	1.787	1.824	0.050	0.028	1.811	1.788	1.836	1.812	0.024	0.013	1.760	1.816	1.835	1.804	0.039	0.022
15	2.236	2.027	2.162	2.142	0.106	0.050	2.066	x	x	2.066	-	-	2.160	2.088	2.124	2.124	0.036	0.017
16	-	-	-	-	-	-	-	-	-	-	-	-	-	-	-	-	-	-
17	-	-	-	-	-	-	-	-	-	-	-	-	-	-	-	-	-	-

- = not measured
* = not recorded because of technical problems
x = not determinable because of peculiarities of curve shape

Table S8. Maximal Adaptive Force of hip flexors.

Values of maximal Adaptive Force (AF_{max}) in N for all measurements (M1, M2, M3) of each patient (ID) for each timepoint (pre = Long COVID state prior to first treatment, post = immediately after first treatment, end = at end measurements). Arithmetic mean (M), standard deviation (SD) and coefficient of variation (CV) for the trials of each patient at each timepoint are given.

	**pre**						**post**						**end**						
ID	M1	M2	M3	M	SD	CV	M1	M2	M3	M	SD	CV	M1	M2	M3	M4	M	SD	CV
1	190.687	193.485	-	192.086	1.978	0.010	181.138	179.044	-	180.091	1.481	0.008	174.927	175.502	-	-	175.215	0.407	0.002
2	130.928	131.474	134.754	132.385	2.069	0.016	210.627	203.642	-	207.135	4.939	0.024	186.797	173.188	190.979	-	183.655	9.302	0.051
3	190.825	218.491	215.530	208.282	15.191	0.073	194.702	232.720	202.597	210.006	20.063	0.096	206.052	211.090	199.681	-	205.608	5.717	0.028
4	181.886	178.878	197.575	186.113	10.040	0.054	195.868	198.146	190.611	194.875	3.864	0.020	121.949	178.839	220.755	-	173.848	49.592	0.285
5	257.852	273.705	240.263	257.273	16.729	0.065	253.944	261.835	247.032	254.270	7.407	0.029	262.595	245.206	260.504	-	256.102	9.494	0.037
6	121.252	144.685	139.392	135.110	12.289	0.091	106.696	145.836	148.195	133.576	23.308	0.174	148.527	166.437	169.670	-	161.545	11.389	0.071
7	126.986	134.309	139.207	133.501	6.150	0.046	129.585	149.176	161.268	146.676	15.989	0.109	143.310	148.512	145.157	-	145.660	2.637	0.018
8	158.117	166.403	170.917	165.146	6.492	0.039	149.226	153.522	157.903	153.550	4.339	0.028	163.388	180.739	158.163	-	167.430	11.818	0.071
9	58.901	67.016	76.214	67.377	8.662	0.129	137.119	189.114	161.492	162.575	26.014	0.160	122.054	131.892	147.502	-	133.816	12.833	0.096
10	183.670	172.019	187.917	181.202	8.231	0.045	114.931	92.008	100.883	102.607	11.558	0.113	108.298	85.902	*	-	97.100	15.836	0.163
11	122.854	125.336	-	124.095	1.755	0.014	108.527	101.918	-	105.223	4.673	0.044	72.541	-	-	-	72.541	-	-
12	184.272	182.435	176.429	181.045	4.102	0.023	154.734	207.600	225.598	195.977	36.834	0.188	229.648	229.595	243.918	238.296	235.364	7.017	0.030
13	155.875	164.593	-	160.234	6.165	0.038	191.509	198.767	-	195.138	5.132	0.026	168.483	173.268	182.626	-	174.792	7.194	0.041
14	173.436	150.551	150.800	158.262	13.141	0.083	137.545	167.652	180.070	161.756	21.867	0.135	157.145	161.688	174.557	-	164.463	9.032	0.055
15	237.431	276.884	227.948	247.421	25.953	0.105	*	211.923	205.540	208.732	4.513	0.022	206.716	196.877	249.748	-	217.780	28.119	0.129
16	257.821	252.178	253.068	254.356	3.034	0.012	218.251	228.159	214.061	220.157	7.240	0.033	225.236	225.332	223.561	-	224.710	0.996	0.004
17	184.637	196.784	*	190.711	8.589	0.045	140.133	153.240	168.789	154.054	14.345	0.093	153.309	188.861	173.837	-	172.002	17.847	0.104

- = not measured

* = not recorded because of technical problems

Table S9. Maximal isometric Adaptive Force of hip flexors.

Values of maximal isometric Adaptive Force ($AFiso_{max}$) in N for all measurements (M1, M2, M3) of each patient (ID) for each timepoint (pre = Long COVID state prior to first treatment, post = immediately after first treatment, end = at end measurements). Arithmetic mean (M), standard deviation (SD) and coefficient of variation (CV) for the trials of each patient at each timepoint are given.

	**pre**						**post**						**end**						
ID	M1	M2	M3	M	SD	CV	M1	M2	M3	M	SD	CV	M1	M2	M3	M4	M	SD	CV
1	136.220	128.938	-	132.579	5.149	0.039	181.138	179.044	-	180.091	1.481	0.008	174.927	175.502	-	-	175.215	0.407	0.002
2	100.991	32.184	82.854	72.010	35.662	0.495	210.627	203.642	-	207.135	4.939	0.024	186.797	173.188	190.979	-	183.655	9.302	0.051
3	x	71.326	51.759	61.543	13.836	0.225	194.702	232.720	202.597	210.006	20.063	0.096	206.052	211.090	199.681	-	205.608	5.717	0.028
4	104.012	97.846	49.979	83.946	29.577	0.352	195.868	198.146	190.611	194.875	3.864	0.020	121.949	178.839	220.755	-	173.848	49.592	0.285
5	173.071	150.240	135.084	152.798	19.122	0.125	253.944	261.835	247.032	254.270	7.407	0.029	262.595	245.206	260.504	-	256.102	9.494	0.037
6	66.293	36.976	41.275	48.181	15.832	0.329	106.696	145.836	148.195	133.576	23.308	0.174	148.527	166.437	169.670	-	161.545	11.389	0.071
7	68.997	84.711	66.723	73.477	9.795	0.133	118.491	149.176	161.268	142.978	22.052	0.154	143.310	148.512	145.157	-	145.660	2.637	0.018
8	99.431	94.852	80.659	91.647	9.788	0.107	149.226	153.522	150.197	150.982	2.253	0.015	155.587	180.739	158.163	-	164.830	13.838	0.084
9	24.605	31.029	26.460	27.365	3.306	0.121	137.119	189.114	161.492	162.575	26.014	0.160	122.054	131.892	147.502	-	133.816	12.833	0.096
10	27.895	45.618	82.474	51.996	27.843	0.535	97.543	87.547	100.883	95.324	6.939	0.073	108.298	85.902	*	-	97.100	15.836	0.163
11	45.215	75.962	-	60.589	21.741	0.359	108.527	101.918	-	105.223	4.673	0.044	72.541	-	-	-	72.541	-	-
12	83.265	89.913	106.628	93.269	12.038	0.129	154.734	207.600	225.598	195.977	36.834	0.188	229.648	229.595	243.918	238.296	235.364	7.017	0.030
13	53.245	96.428	-	74.837	30.535	0.408	175.219	163.788	-	169.504	8.083	0.048	168.483	173.268	182.626	-	174.792	7.194	0.041
14	73.948	37.964	89.363	67.092	26.377	0.393	137.545	167.652	176.918	160.705	20.585	0.128	157.145	161.688	174.557	-	164.463	9.032	0.055
15	134.155	175.926	190.107	166.729	29.088	0.174	*	211.923	205.540	208.732	4.513	0.022	206.716	196.877	249.748	-	217.780	28.119	0.129
16	120.478	182.086	172.154	158.239	33.077	0.209	218.251	228.159	214.061	220.157	7.240	0.033	225.236	225.332	223.561	-	224.710	0.996	0.004
17	74.856	94.718	*	84.787	14.045	0.166	140.133	153.240	168.789	154.054	14.345	0.093	153.309	188.861	173.837	-	172.002	17.847	0.104

- = not measured
* = not recorded because of technical problems
x = not determinable because of peculiarities of curve shape

Table S10. Adaptive Force at onset of oscillations of hip flexors.

Values of Adaptive Force at onset of oscillations (AFosc) in N for all measurements (M1, M2, M3) of each patient (ID) for each timepoint (pre = Long COVID state prior to first treatment, post = immediately after first treatment, end = at end measurements). Arithmetic mean (M), standard deviation (SD) and coefficient of variation (CV) for the trials of each patient at each timepoint are given.

	**pre**						**post**						**end**						
ID	M1	M2	M3	M	SD	CV	M1	M2	M3	M	SD	CV	M1	M2	M3	M4	M	SD	CV
1	190.687	190.582	-	190.635	0.074	0.000	132.284	117.013	-	124.649	10.798	0.087	159.098	129.240	-	-	144.169	21.113	0.146
2	130.928	131.474	134.754	132.385	2.069	0.016	111.244	154.154	-	132.699	30.342	0.229	153.286	129.483	160.946	-	147.905	16.407	0.111
3	x	197.620	168.684	183.152	20.461	0.112	135.928	192.746	175.909	168.194	29.184	0.174	137.997	138.722	154.547	-	143.755	9.353	0.065
4	175.559	178.878	176.386	176.941	1.728	0.010	141.785	142.896	143.373	142.685	0.815	0.006	53.816	70.962	108.839	-	77.872	28.155	0.362
5	225.913	265.767	232.807	241.496	21.300	0.088	159.465	226.124	180.979	188.856	34.020	0.180	173.746	203.297	198.222	-	191.755	15.801	0.082
6	121.252	144.685	139.392	135.110	12.289	0.091	66.599	76.720	61.751	68.357	7.638	0.112	118.746	108.636	110.909	-	112.764	5.304	0.047
7	118.959	134.309	139.207	130.825	10.564	0.081	84.647	90.500	102.183	92.443	8.928	0.097	112.844	123.755	104.218	-	113.606	9.791	0.086
8	141.731	159.700	170.917	157.449	14.723	0.094	111.655	129.339	117.462	119.485	9.014	0.075	101.311	126.320	118.279	-	115.303	12.767	0.111
9	57.873	65.367	76.214	66.485	9.221	0.139	70.876	68.758	49.005	62.880	12.062	0.192	68.419	60.524	80.718	-	69.887	10.177	0.146
10	177.655	163.525	181.114	174.098	9.318	0.054	76.957	60.142	66.603	67.901	8.482	0.125	78.356	29.286	*	-	53.821	34.698	0.645
11	116.039	117.759	-	116.899	1.216	0.010	77.611	61.146	-	69.379	11.643	0.168	29.859	-	-	-	29.859	-	-
12	182.815	181.119	175.645	179.860	3.747	0.021	92.277	129.901	132.626	118.268	22.550	0.191	112.546	143.518	103.636	133.196	123.224	18.339	0.149
13	155.875	164.593	-	160.234	6.165	0.038	162.806	163.385	-	163.096	0.409	0.003	122.255	113.404	147.335	-	127.665	17.600	0.138
14	173.436	150.551	148.436	157.474	13.864	0.088	84.136	75.950	65.697	75.261	9.239	0.123	83.087	54.991	84.139	-	74.072	16.533	0.223
15	193.910	214.538	205.903	204.784	10.359	0.051	*	152.646	155.572	154.109	2.069	0.013	114.281	123.114	113.826	-	117.074	5.236	0.045
16	257.821	215.623	253.068	242.171	23.113	0.095	106.130	134.636	156.566	132.444	25.289	0.191	152.184	135.157	152.103	-	146.481	9.807	0.067
17	184.637	196.784	*	190.711	8.589	0.045	89.582	113.749	94.616	99.316	12.751	0.128	86.605	62.087	96.651	-	81.781	17.780	0.217

- = not measured
* = not recorded because of technical problems
x = not determinable because of peculiarities of curve shape

Table S11. Slope of force increase for hip flexors.

Logarithmic slope of force increase during AF measurements in lg(N/s)) for all measurements (M1, M2, M3) of each patient (ID) for each timepoint (pre = Long COVID state prior to first treatment, post = immediately after first treatment, end = at end measurements). Arithmetic mean (M), standard deviation (SD) and coefficient of variation (CV) for the trials of each patient at each timepoint are given.

	pre						post						end						
ID	M1	M2	M3	M	SD	CV	M1	M2	M3	M	SD	CV	M1	M2	M3	M4	M	SD	CV
1	2.180	2.030	-	2.105	0.106	0.050	1.749	1.765	-	1.757	0.012	0.007	1.997	x	-	-	1.997	#DIV/0!	#DIV/0!
2	1.774	1.916	1.870	1.854	0.073	0.039	1.707	1.969	-	1.838	0.186	0.101	1.905	1.838	1.904	-	1.882	0.038	0.020
3	1.587	1.657	1.811	1.685	0.115	0.068	1.982	1.866	2.084	1.977	0.109	0.055	1.913	1.863	1.693	-	1.823	0.115	0.063
4	1.877	1.746	1.835	1.819	0.067	0.037	1.996	1.983	2.019	1.999	0.018	0.009	1.856	x	2.020	-	1.938	0.116	0.060
5	2.059	2.189	2.113	2.120	0.066	0.031	2.171	2.228	2.175	2.191	0.032	0.014	2.037	2.159	2.035	-	2.077	0.071	0.034
6	1.688	1.669	1.572	1.643	0.062	0.038	1.692	1.788	1.894	1.791	0.101	0.056	1.806	1.739	1.790	-	1.778	0.035	0.020
7	1.896	1.748	1.917	1.854	0.092	0.050	1.909	2.063	2.047	2.006	0.085	0.042	1.925	1.779	1.945	-	1.883	0.091	0.048
8	1.788	1.776	1.682	1.749	0.058	0.033	2.050	2.068	2.011	2.043	0.029	0.014	2.136	1.917	2.017	-	2.023	0.110	0.054
9	1.357	1.419	1.469	1.415	0.056	0.040	1.560	1.663	1.535	1.586	0.068	0.043	1.367	1.525	1.634	-	1.509	0.135	0.089
10	1.922	1.566	1.806	1.765	0.182	0.103	1.930	1.829	1.876	1.878	0.050	0.027	1.867	1.648	*	-	1.758	0.155	0.088
11	1.750	1.815	-	1.783	0.046	0.026	1.894	1.846	-	1.870	0.034	0.018	x	-	-	-	-	-	-
12	1.930	2.076	2.107	2.038	0.095	0.047	2.017	1.901	2.054	1.991	0.080	0.040	1.847	2.205	1.960	1.823	1.959	0.175	0.089
13	1.625	2.039	-	1.832	0.292	0.160	1.839	2.237	-	2.038	0.282	0.138	1.835	2.023	1.877	-	1.912	0.099	0.052
14	1.824	1.760	1.846	1.810	0.044	0.025	1.821	1.821	1.888	1.843	0.039	0.021	1.897	x	1.835	-	1.866	0.044	0.024
15	2.122	2.048	2.083	2.084	0.037	0.018	*	2.175	2.033	2.104	0.100	0.048	2.014	2.001	2.005	-	2.007	0.007	0.003
16	2.054	2.112	1.763	1.976	0.187	0.095	x	1.995	x	1.995	-	-	x	2.029	2.084	-	2.056	0.039	0.019
17	1.845	1.953	*	1.899	0.076	0.040	x	1.815	1.865	1.840	0.035	0.019	1.662	1.703	1.955	-	1.773	0.159	0.089

- = not measured
* = not recorded because of technical problems
x = not determinable because of peculiarities of curve shape
